# CD44‐Binding Peptide‐Functionalized Antibiofouling Polymer Surface for High‐Performance Separation of Human Mesenchymal Stromal Cells

**DOI:** 10.1002/cbic.202500822

**Published:** 2026-02-12

**Authors:** Moe Kato, Tadashi Nakaji‐Hirabayashi, Kazuaki Matsumura, Chiaki Yoshikawa, Yuki Usui, Takahiro Kishioka, Taito Nishino

**Affiliations:** ^1^ Graduate School of Innovative Life Science University of Toyama Toyama Japan; ^2^ Faculty of Engineering Academic Assembly University of Toyama Toyama Japan; ^3^ Graduate School of Science and Engineering University of Toyama Toyama Japan; ^4^ Research Center for Macromolecules and Biomaterials National Institute for Materials Science (NIMS) Tsukuba Japan; ^5^ School of Materials Science Japan Advanced Institute of Science and Technology Nomi Japan; ^6^ Materials Research Laboratories Nissan Chemical Corporation Toyama Japan; ^7^ Biological Research Laboratories Nissan Chemical Corporation Shiraoka Japan

**Keywords:** antifouling surface, cell separation, human mesenchymal stem cell, label‐free purification, peptide‐functionalized polymer, regenerative medicine, zwitterionic polymer

## Abstract

Cell transplantation therapy is a promising strategy for next‐generation regenerative medicine. However, its clinical application is limited by the absence of safe and label‐free methods for obtaining pure populations of functional cells. This study reports a peptide‐functionalized zwitterionic polymer system designed for the selective and noninvasive isolation of human mesenchymal stem cells (hMSCs) from heterogeneous cell populations. The substrate was modified with poly(CMBMAm‐*co*‐PGMAn‐*co*‐MPTMS1) (PC*_m_* P_*n*_M_1_), which was synthesized via free radical polymerization, and subsequently conjugated with a CD44‐binding peptide (CD44BP). Among the compositions examined, the PC_7_P_2_M_1_–CD44BP surface exhibited superior antifouling properties, effectively suppressing nonspecific protein adsorption and adhesion of nontarget cells while selectively capturing hMSCs. A column packed with PC_7_P_2_M_1_–CD44BP–modified silica microparticles successfully isolated high‐purity hMSCs from mixed cell suspensions within 35 min. Flow cytometry confirmed that the cells eluted later exhibited higher CD44 and CD105 expression, indicating separation based on antigen expression. The isolated hMSCs maintained proliferation and differentiation capacities equivalent to those of the preseparated cells, demonstrating that this device preserved cell functionality. This peptide–polymer hybrid column provides a simple and clinically adaptable platform for safe, label‐free purification of hMSCs intended for cell transplantation therapy.

## Graphical abstract

A peptide‐functionalized zwitterionic polymer enables label‐free and noninvasive isolation of human mesenchymal stem cells (hMSCs). The antifouling polymer surface selectively captured hMSCs based on CD44 recognition, achieving high‐purity separation while preserving cell viability and differentiation potential for safe cell transplantation applications.

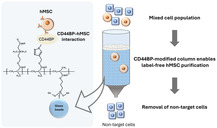

## 1 Introduction

Cell transplantation therapy is anticipated to become the cornerstone of next‐generation medical interventions, and numerous studies are actively progressing toward its practical implementation. However, this therapy requires overcoming several challenges, including the establishment of a stable cell supply [1], precise control of cellular behavior [2], and safety assurance [1]. Among these, the development of techniques for the selective isolation and purification of target cells is particularly critical.

The candidate sources of transplantable cells include autologous and allogeneic cells [3]. However, regardless of the source, the collected cell populations consisted of multiple cell types, making the isolation of specific target cells difficult. Several studies have reported that undesired cells are associated with carcinogenesis and reduced differentiation efficiency. Moreover, the inclusion of undifferentiated cells within differentiated cell populations increases the risk of carcinogenesis, highlighting the essential need for selective cell isolation, not only during tissue engineering but also during transplantation. In this context, the development of reliable techniques for the isolation and recovery of specific cell types is crucial [4], and continued technological advancements are indispensable for the successful realization of cell transplantation therapy [5].

Currently, techniques such as fluorescence‐activated cell sorting (FACS) [6], magnetic‐activated cell sorting (MACS) [7], and density gradient centrifugation [8] are being widely used to isolate specific cell types. Although these methods are well‐established and commonly used in research, challenges remain regarding their safety and procedural complexity when applied to the separation of transplantable cell sources. For instance, FACS can induce cytotoxicity owing to residual fluorescent labeling agents on the cell surface and the mechanical stress exerted by fluid flow [9]. Similarly, MACS is associated with residual labeling agents, and the removal of the magnetic beads is highly complex. In the case of density gradient centrifugation, concerns persist regarding residual solvents, such as Ficoll and Percoll. Ficoll is cytotoxic and unsuitable for clinical use, whereas Percoll, although less toxic, requires caution when used in clinical applications. Therefore, although the existing separation methods offer high accuracy and versatility, their suitability for clinical‐grade cell separation remains limited.

Considering these limitations, we aimed to develop a device capable of separating specific cell types from heterogeneous cell populations with minimal invasiveness and a simple operation. Considering practical applications, the device was designed for convenient operation on a clean bench, eliminating the need for large‐scale equipment. Our research group previously demonstrated that coating surfaces with zwitterionic polymers effectively inhibited nonspecific proteins and cell adhesion [10]. Recent studies have highlighted the advantages of zwitterionic polymer‐based surface coatings, particularly their long‐term stability and enhanced antibiofouling performance. For instance, self‐renewing zwitterionic silicone hybrid coatings have been reported to provide multimodal bacterial resistance and robust antibiofouling properties [11]. These advancements underscore the growing relevance of zwitterionic materials in preventing undesired bioadhesion and support the rationale for employing such materials in the development of practical cell separation platforms. Based on this foundation, various functional materials have been developed for this purpose. Additionally, we have developed advanced device designs that enable the immobilization of proteins and oligopeptides onto substrates while preserving their biological activity, thereby promoting the adhesion and functionality of target cells [12].

Based on these insights, we developed a device capable of selectively capturing specific cell types. Human mesenchymal stromal/stem cells (hMSCs) were selected as the target cell type because they are among the most promising cells for clinical applications. Using phage display technology, our research group previously identified a peptide sequence, QQGWFPGAG (CD44‐binding peptide, CD44BP), that selectively binds to the CD44 cell membrane receptor antigen in hMSCs [13]. By employing this peptide to interact with antigens on the surface of target cells, we hypothesized that highly precise and noninvasive cell separation could be achieved. Furthermore, to minimize the nonspecific adhesion of unintended cells and proteins [4], which is often observed in separation methods utilizing molecular interactions, we introduced zwitterionic polymers to suppress random adsorption.

In this study, we developed microparticle carriers capable of selectively capturing hMSCs by incorporating a carboxymethyl betaine (CMBMA) polymer [10], which suppresses nonspecific protein and cell adhesion, and introducing CD44BP into the polymer side chains. Subsequently, we constructed a cell separation column packed with silica microparticles coated with a CD44BP‐functionalized antibiofouling polymer [13]. The selective separation efficiency of hMSCs using this column was comprehensively evaluated along with the maintenance of hMSC functionality after separation.

## 2 Materials and Methods

### 2.1 Synthesis of Ternary Copolymer: Poly(carboxymethylbetaine‐*co*‐propargyl methacrylate‐*co*‐3‐methacryloyloxypropyl trimethoxysilane)

**SCHEME 1:**
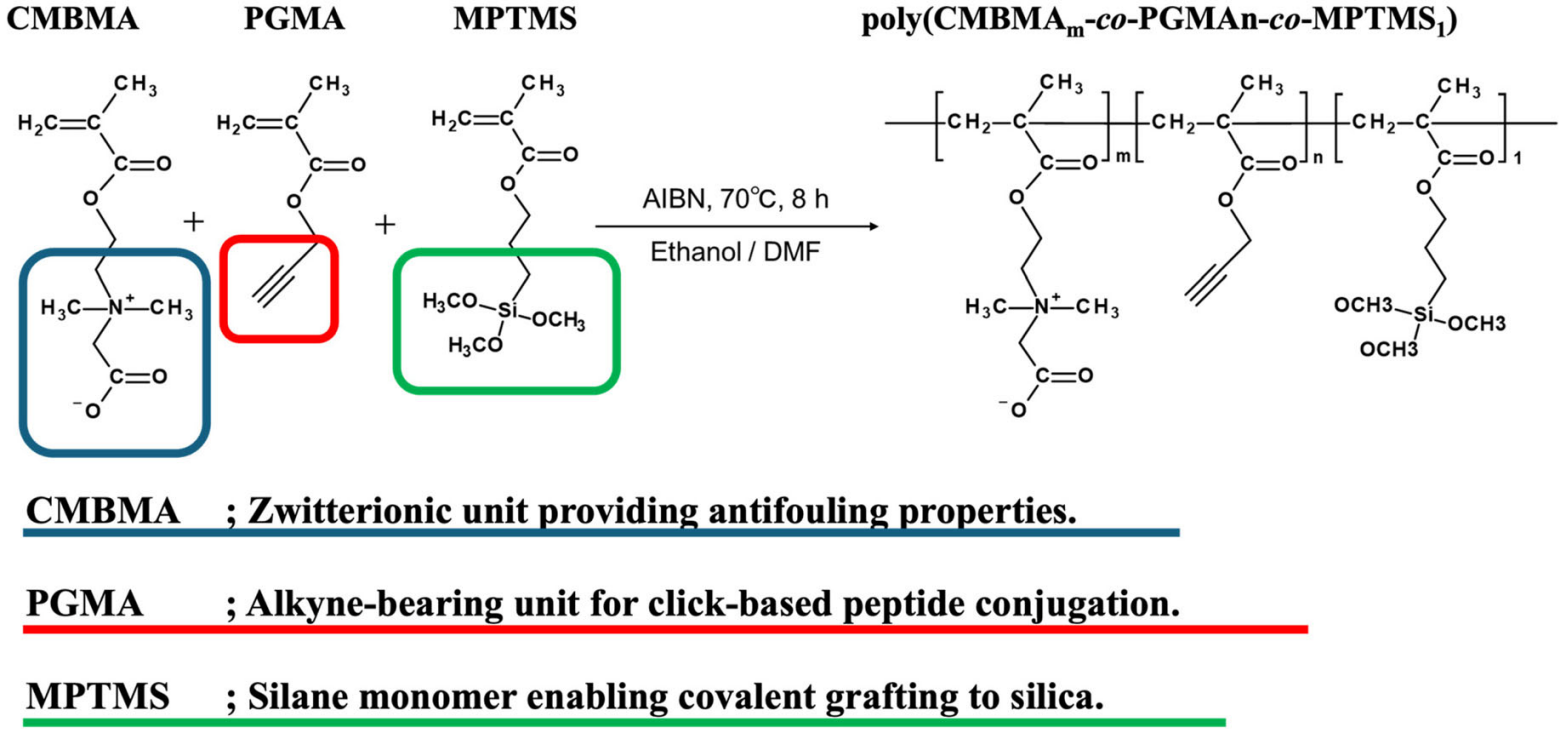
Synthesis of a functional copolymer composed of CMBMA (antifouling unit), PGMA (alkyne unit for click‐mediated peptide conjugation), and MPTMS (silane monomer for anchoring to silica substrates).

A mixed solvent of ethanol (EtOH) and N, N‐dimethylformamide (DMF), dehydrated using 3 Å molecular sieves, was prepared by dissolving carboxy‐N, N‐dimethyl‐N‐(2′‐methacryloyloxyethyl) methanaminium inner salt (CMBMA; Osaka Organic Chemical Industry Co., Ltd., Osaka, Japan), propargyl methacrylate (PGMA; Hydrus Chemical Inc., Tokyo, Japan), and 2,2′‐azobisisobutyronitrile (AIBN; FUJIFILM Wako Pure Chemical Co., Ltd., Osaka, Japan) at a concentration of 2.19 × 10^−2^ M. The total monomer concentration for the polymerization reaction was fixed at 0.5 M. To remove dissolved oxygen, the mixture was purged with nitrogen gas for 20 min. Subsequently, 3‐(trimethoxysilyl) propyl methacrylate (MPTMS; Tokyo Chemical Industry Co., Ltd., Tokyo, Japan) was added, followed by an additional 10‐min nitrogen purge. The react ion vessel was then sealed and maintained at 70°C for 8 h (Scheme 1). To clarify the design rationale for the copolymer, the roles of each monomer are summarized as follows: CMBMA possesses a zwitterionic structure and exhibits strong antibiofouling properties by suppressing nonspecific proteins and cell adhesion. PGMA, which contains a terminal alkyne group, serves as a reactive site for the introduction of the CD44‐binding peptide (CD44BP) into the polymer side chains. In this study, CD44BP modified with an azide group was used, and the peptide was covalently immobilized through a click reaction between the azide and alkyne groups, forming a stable 1,2,3‐triazole linkage under mild and efficient reaction conditions. MPTMS acts as a silane coupling monomer, enabling the covalent attachment of the copolymer to the silica substrate through hydrolyzable alkoxysilane groups. By combining these three monomers, the copolymer simultaneously achieved antifouling capability, click‐chemistry‐based peptide immobilization, and strong anchoring to the substrate, thereby creating a functional surface well suited for the selective isolation of hMSCs. Table 1 summarizes the polymerization conditions for each composition ratio and molecular weight of the synthesized polymers, which were determined using gel permeation chromatography (GPC). The polymers obtained with various composition ratios, poly(CMBMA*_m_*‐*co*‐PGMAn‐*co*‐MPTMS_1_), are abbreviated as PC*_m_* P*_n_* M_1_.

**TABLE 1:** Reaction conditions and characterization of poly(CMBMA*_m_*‐*co*‐PGMA*_n_*‐*co*‐MPTMS_1_) (PC*_m_* P*_n_* M_1_).^a^

Sample	Feeding ratio^a^ (CMBMA : PGMA : MPTMS)	Solvent (EtOH/DMF)	M_w_ (M_w_/M_n_)^b^
PC_9_P_0_M_1_	9 : 0 : 1	10/0	3.29 × 10^4^ (3.81)
PC_8_P_1_M_1_	8 : 1 : 1	9.5/0.5	2.53 × 10^4^ (3.28)
PC_7_P_2_M_1_	7 : 2 : 1	7.5/2.5	2.29 × 10^4^ (3.15)
PC_6_P_3_M_1_	6 : 3 : 1	7.5/2.5	2.00 × 10^4^ (2.95)
PC_5_P_4_M_1_	5 : 4 : 1	7.5/2.5	1.66 × 10^4^ (3.26)

a The composition of MPTMS was fixed at 10%. This ratio was optimized for achieving adequate surface coverage and enhancing the antibiofouling performance of CMBMA [10].

b The average molecular weight and polydispersity index of PC*_m_* P_*n*_M_1_ were determined by GPC. Solvent: methanol/DMF mixture (3:1). Column: Wako Beads G‐50. Standard: PMMA.

### 2.2 Synthesis of CD44‐Binding Peptide

Azidohomoalanine (Aha)‐terminated CD44BP was synthesized via solid‐phase peptide synthesis using an Rotary Shaker N‐500 (Kokusan Chemical Co., Ltd.). Fmoc‐NH‐SAL‐PEG resin (0.21 mmol/g; Watanabe Chemical Industries Ltd., Hiroshima, Japan) was washed with DMF and methanol, followed by swelling in a solvent containing 25% dimethyl sulfoxide (DMSO) in DMF for 30 min. Subsequently, the resin‐bound amino acid Fmoc‐protecting groups were removed by washing with DMF and stirring the resin in 20% piperidine/DMF for 30 min. The resin was then washed again with DMF and methanol and resuspended in 25% DMSO/DMF. Fmoc‐amino acids (0.63 mmol; Watanabe Chemical Industries Ltd.) was coupled to the resin by the addition of 4‐(4,6‐dimethoxy‐1,3,5‐triazin‐2‐yl)‐4‐methylmorpholinium chloride (1.05 mmol) and 4‐methylmorpholine (0.42 mmol), followed by stirring to promote coupling. Peptide cleavage from the resin was achieved by stirring the resin in a solvent mixture containing 9.5 mL of trifluoroacetic acid (TFA), 0.25 mL of triisopropylsilane, and 0.25 mL of water per 1 g of Fmoc‐NH‐SAL‐PEG resin for 3 h. After cleavage, the peptide was precipitated by cooling with diethyl ether and collected by centrifugation (2380 × g, 30 min, −4°C) to remove residual ether. The peptide was redissolved in water, and the resin was removed by filtration. The filtrate was flash‐frozen in liquid nitrogen and lyophilized using a small freeze dryer (FDS‐1000, Tokyo Rikakikai Co., Ltd., Tokyo, Japan).

Amino acid sequences of the synthesized peptides are summarized in Table 2. To evaluate the effect of linker length on the cell capture efficiency, three CD44BPs with different linker lengths were synthesized. Additionally, to confirm that cellular capture was specifically mediated by CD44BP, inhibition experiments were performed using freely available (nonimmobilized) CD44BP. Therefore, a CD44BP variant without Aha was synthesized for comparison.

**TABLE 2:** Amino acid sequences of CD44‐binding peptides.

Peptide	Amino acid sequence
CD44BP‐Aha(L0)	Gln–Gln–Gly–Trp–Phe–Pro–Aha
CD44BP‐Aha(L3)	Gln–Gln–Gly–Trp–Phe–Pro–Gly–Ala–Gly–Aha
CD44BP‐Aha(L14)	Gln–Gln–Gly–Trp–Phe–Pro–(Gly–Ala)_7_–Aha
CD44BP(L3)	Gln–Gln–Gly–Trp–Phe–Pro–Gly–Ala–Gly

### 2.3 Construction of Surface Modified With Ternary Copolymer Immobilized With CD44BP

Glass substrates (26 × 20 mm; ASONE Co., Ltd., Tokyo, Japan) and silicon wafers (20 × 20 mm; Matsuzaki Seisakusyo Co., Ltd., Shimane, Japan) were immersed in a piranha solution (7:3 mixture of sulfuric acid and hydrogen peroxide) for 30 min. The substrates were then rinsed thoroughly with distilled water and methanol and dried under an air stream. The cleaned substrates were subsequently immersed in a 1% w/v solution of PC*_m_* P*_n_* M_1_ at room temperature for 24 h to modify the polymer surface via a silane coupling reaction. After the modification, the substrates were removed from the solution, rinsed with ethanol and methanol, and dried.

The PC*_m_* P*_n_* M_1_‐modified substrates were then immersed in an aqueous solution containing CD44BP‐Aha (9.32 × 10^−4^ mol/mL), copper sulfate (9.32 × 10^−5^ mol/mL), and sodium L‐ascorbate (9.32 × 10^−5^ mol/mL), and reacted at room temperature for 24 h. This step immobilized CD44BP onto the polymer surface through a copper(I)‐catalyzed azide–alkyne cycloaddition “click” reaction between the alkyne groups of the polymer side chains and the azide terminus of the peptide (Scheme S1). After the reaction, the modified substrates were thoroughly washed with distilled water and dried under a stream of nitrogen gas.

The surface modification of nonporous silica microspheres (250–350 µm; Fuji Manufacturing Co., Ltd., Tokyo, Japan) used in the cell separation experiments was performed as follows. The silica microspheres were immersed in the piranha solution for 30 min in a glass centrifuge tube and then thoroughly rinsed with distilled water until the pH reached 7.0. Subsequently, the solution was replaced with dehydrated ethanol and the microspheres were agitated at room temperature for 24 h in a 2 w/v% PC*_m_* P*_n_* M_1_ solution. After washing with ethanol and distilled water, CD44BP‐Aha solution (1 mg/mL) was added, and the suspension was agitated at room temperature for 24 h. After the reaction, the particles were rinsed thoroughly with distilled water to obtain PC*_m_* P*_n_* M_1_–CD44BP–modified silica microspheres (Scheme S2).

### 2.4 Characterization of PC*_m_* P*_n_* M_1_

The molecular weight of PC*_m_* P*_n_* M_1_, prepared as a 10% (w/v) solution, was determined using GPC (Agilent 1260 Infinity; Agilent Technologies Japan, Ltd., Tokyo, Japan). Wakopak Wakobeads‐G‐50 columns (FUJIFILM Wako Pure Chemical Co., Ltd.) were used for chromatographic separation. The eluent consisted of a mixture of DMF) and methanol (1:3, v/v) with a flow rate of 1.0 mL/min. The polymers were detected using a refractive‐index detector (Agilent Technologies, Japan, Ltd.). poly(methyl methacrylate) (PMMA; Standard M‐75, Showa Denko K.K., Tokyo, Japan) was used as the calibration standard. Data analysis was conducted using an SIC µ7 Plus system (SYSTEM INSTRUMENTS Co., Ltd., Tokyo, Japan).

### 2.5 Characterization of CD44BP

Circular dichroism (CD) spectra of the synthesized peptides were obtained using a circular dichroism spectropolarimeter (J‐820; JASCO Corporation, Tokyo, Japan). Measurements were performed with a sensitivity of 100 mdeg over a wavelength range of 300–180 nm, at a scanning speed of 100 nm/min, with data accumulated over eight scans at 37°C. A quartz cell with a 0.2 cm path length of used. Data analysis was conducted using the JASCO Spectrum Manager software (JASCO Corporation, Tokyo, Japan). The mean residue ellipticity ([*θ*], deg·cm^2^·dmol^−1^) was calculated according to Equation (1), where *θ* represents ellipticity (mdeg), *l* is the path length (cm), *c* is the peptide concentration (mol/L), and *n* is the number of residues in the peptide [14].

(1) [θ]=θ/10lcn

Liquid chromatography–mass spectrometry (LC–MS) of the synthesized peptides was performed using a Shimadzu ProminenceThermo LXQ system. The mobile phase consisted of acetonitrile: water (1:1, v/v) containing 0.1% TFA. The peptide solution (1 mg/mL) was eluted at a flow rate of 1.0 mL/min using a GL Science Inert Sustain C18 column (5 µm). Data analysis was performed using Xcalibur software (Thermo Fisher Scientific Inc.).

### 2.6 Wettability of Ternary Polymer‐ and CD44BP‐Carried Ternary Polymer‐Modified Surfaces

To evaluate the wettability of the PC*_m_* P_*n*_M_1_– and PC_*m*_P_*n*_M_1_–CD44BP‐modified surfaces, contact angle measurements were conducted using a contact angle meter (DMs‐401; Kyowa Interface Science Co., Ltd., Saitama, Japan). A 1.0 μL droplet of distilled water was placed on the substrate at room temperature, and the contact angle was measured after 30 s using the *θ*/2 method. Ten measurements were performed for each modified surface and the mean value was recorded as the representative static contact angle. In addition, dynamic contact angle measurements were performed to assess the time‐dependent changes in surface wettability. A 1.0 μL water droplet was placed on the surface, and the contact angle was recorded every 5 s for 10 min. Dynamic contact angle profiles were obtained from ten independent measurements for each sample [15].

### 2.7 Characterization of Ternary Polymer‐ and CD44BP‐Carried Ternary Polymer‐Modified Surface

Each modified surface was characterized using X‐ray photoelectron spectroscopy (XPS) with an ESCALAB 250Xi instrument (Thermo Fisher Scientific K.K., Waltham, MA, USA). Measurement conditions included a sample angle of 0°, an X‐ray incidence angle of 32°, and a detector reflection angle of 90°, with a 200 μm X‐ray spot size. The binding energies of the detected elements were as follows: C1s, 280–300 eV; O1s, 527–545 eV; N1s, 392–410 eV; and Si2p, 95–110 eV. Spectral analysis was conducted using Advantage software (version 5.1; Thermo Fisher Scientific Inc.).

Attenuated total reflection Fourier transform infrared (ATR–FTIR) spectroscopy was performed using an infrared spectrophotometer (Nicolet iS5 FT‐IR; Thermo Fisher Scientific, Inc.). Silicon wafers were used as the baseline reference, and absorption spectra were recorded in the range of 400–4000 cm^−1^ with 512 scans. Data analysis was conducted using the OMNIC software (Thermo Fisher Scientific Inc.).

The film thickness on the substrate surface in the dry state was determined using an automatic ellipsometer (MARY‐102, Five Lab Co., Ltd., Saitama, Japan) based on the ellipsometry principle. A 632.8 nm He–Ne laser was employed as the light source. For the polymer layer measurements, the refractive index of PMMA was set to 1.489, and that of the peptide layer was set to 1.459.

The surfaces of the polymer‐modified and peptide‐functionalized substrates were examined using a tabletop scanning electron microscope (SEM) (Miniscope TM4000Plus II, Hitachi High‐Tech Corp., Tokyo, Japan; 2021). The acceleration voltage was set to 10 kV. The SEM images were acquired at two magnifications, 100× and 5000×, to evaluate the overall surface uniformity and fine microstructural features.

The amount of peptide loaded onto the substrate was quantified using the bicinchoninic acid (BCA) assay. The dry weights of the polymer‐modified substrates (26 mm × 38 mm) and peptide‐carrying polymer‐modified substrates (26 mm × 38 mm) were measured. After pretreatment with Dulbecco's phosphate‐buffered saline (PBS, without Ca^2+^/Mg^2+^), 400 μL of BCA reagent was applied to each substrate and incubated for 2 h at 37°C. The absorbance at 570 nm was measured using a microplate reader (Multiskan JX; Thermo Fisher Scientific Inc., USA). A calibration curve was constructed using standard albumin (BSA) standard solutions.

### 2.8 Antibiofouling Property of Ternary Polymer‐ and CD44BP‐Carried Ternary Polymer‐Modified Surfaces

The amount of adsorbed BSA was quantified using the bicinchoninic acid assay to evaluate the inhibition of protein adsorption on each modified surface. Each polymer‐modified and peptide‐loaded substrate (26 mm × 38 mm) was pretreated with PBS and subsequently incubated with a 2 wt% BSA solution in PBS at room temperature for 2 h. After incubation, the BSA solution was removed and each substrate was washed ten times with PBS to eliminate unbound proteins. A silicon frame cleaned and cut to match the dimensions of the substrate was placed at both ends of the modified substrate. A cleaned glass slide was then placed on top using a silicon frame as the spacer. The gap between the modified and glass substrates was filled with BCA reagent, which served as the colorimetric indicator, and the assembly was incubated at 37°C for 2 h. The absorbance of the resulting solution was measured at 570 nm using a microplate reader (Multiskan JX; Thermo Fisher Scientific Inc.). A calibration curve was generated using standard BSA solutions of known concentrations.

### 2.9 Selectivity of Cell Capture on CD44BP‐Carried Ternary Polymer‐Modified Surfaces

To evaluate the cell capture selectivity of the PC*_m_* P*_n_* M_1_‐and PC*_m_* P*_n_* M_1_–CD44BP‐modified surfaces, hMSCs, which express the CD44 surface antigen, and human embryonic kidney 293 (HEK293) cells, which lack CD44 expression, were seeded onto each modified surface.

Frozen human mesenchymal stem cells (hMSCs; MSC‐R50, Cell No. : HMS0047; RIKEN BioResource Research Center, Japan, passage 2) and HEK293 cells (Research Resource Identifier (RRID): CVCL_0045, passage 157) were thawed and seeded onto 10 cm polystyrene culture dishes, then cultured for 3 days in growth medium containing 10% fetal bovine serum (FBS), 100 U/mL penicillin, and 100 μg/mL streptomycin in Dulbecco's Modified Eagle Medium (DMEM, high glucose) at 37°C and 5% CO_2_. Mouse fibroblast cells (NIH3T3; RRID: CVCL_0594, passage 148) were maintained under similar conditions using Minimum Essential Medium supplemented with 10% FBS, 100 U/mL penicillin, and 100 μg/mL streptomycin at 37°C and 5% CO_2_.Upon reaching 70%–80% confluence, the cells were detached using 0.25% trypsin–1 mM EDTA solution (Nacalai Tesque, Kyoto, Japan) and used for subsequent experiments.

The collected single‐cell suspensions were seeded onto unmodified glass, PC*_m_* P*_n_* M_1_‐modified, or PC*_m_* P*_n_* M_1_–CD44BP‐modified glass substrates (26 mm × 20 mm) at a density of 2.0 × 10^4^ cells/cm^2^. All the substrates were sterilized by immersion in 70% ethanol, air‐dried, and handled aseptically on a clean bench. After 24 h of incubation at 37°C and 5% CO_2_, the cells were rinsed with PBS to remove nonadherent cells. Cell adhesion was observed using a phase‐contrast microscope (IX71; Olympus Corporation, Tokyo, Japan), and the number of adherent cells was quantified by staining the nuclei with Hoechst 33 342.

### 2.10 Construction of Cell‐Separating Column

The silica microspheres functionalized with PC_7_P_2_M_1_–CD44BP‐modified surfaces were sterilized by immersion in 70% ethanol. Within a clean bench, the ethanol was completely replaced with PBS, and the microspheres were packed into a syringe equipped with a 100 μm filter (Cell Fraction Filter Filcon Syringe 100 μm; AS ONE Corporation, Osaka, Japan) to construct the PC_7_P_2_M_1_–CD44BP‐modified column. The column was densely packed with silica microspheres by continuously passing PBS overnight through a peristaltic pump (Quantitative Liquid Feed Pump; Tokyo Rikakikai Co., Ltd., Tokyo, Japan) (Figure S1).

### 2.11 Fluorescent Labeling of Cells for Cell Separation Analysis

To differentiate between HEK293 cells and hMSCs, fluorescent staining was performed using the CellTracker dye (Thermo Fisher Scientific Inc., USA). HEK293 cells were labeled with CellTracker Green CMFDA dye and hMSCs were labeled with CellTracker Orange CMRA dye. Both dyes were dissolved in DMSO to prepare 1.0 mM stock solutions, which were subsequently diluted in serum‐free medium to final concentrations of 3.0 μM (CellTracker Green) and 2.0 μM (CellTracker Orange). After culturing the cells to ≈70%–80% confluence, the HEK293 and hMSC monolayers were rinsed with PBS, and the respective staining solutions were added. Cells were incubated at 37°C and 5% CO_2_ for 30 min. Following incubation, the staining solutions were removed and the cells were recovered by adding DMEM supplemented with FBS and incubating under the same conditions.

### 2.12 Specific Cell Separation

The PC_7_P_2_M_1_–CD44BP‐modified column was first equilibrated with PBS, after which 200 mL of serum‐free DMEM was passed through the column to replace the buffer (Figure S1). Prelabeled HEK293 and hMSC suspensions were prepared, each containing 1.0 × 10^6^ cells. A 1 mL aliquot of the mixed cell suspension was loaded onto the column, followed by perfusion with 50 mL of serum‐free DMEM. The eluate, which immediately flowed out from the bottom of the column, was collected in 1 mL fractions. After collecting 50 mL of the medium, 100 mL of 5 mM EDTA in PBS was introduced into the column to detach weakly adherent cells, which were subsequently recovered. The number of cells in each fraction was determined using a hemocytometer under a fluorescence microscope. Additionally, the cells recovered with EDTA/PBS were subjected to a second round of separation by reloading onto a new column.

Furthermore, to verify that the cell‐separation capability of the PC_7_P_2_M_1_–CD44BP‐modified column originates from the specific interaction between hMSCs and immobilized CD44BP on the silica particle surface, a competitive inhibition experiment was performed by preincubating cells with free CD44BP prior to column loading. This experiment demonstrated that the precontact of hMSCs with free CD44BP attenuated their interaction with the PC_7_P_2_M_1_–CD44BP‐modified column, thereby decreasing the separation efficiency. As described above, a PC_7_P_2_M_1_–CD44BP‐modified column was prepared and suspensions of CellTracker‐labeled HEK293 cells and hMSCs (each 1.0 × 10^6^ cells in 1 mL) were supplemented with 700 nmol/mL free CD44BP. The mixed cell suspension (1 mL) was loaded onto the column, followed by perfusion with 30 mL of a medium containing the same concentration of CD44BP. The eluate was collected in 1 mL fractions. Finally, 20 mL of the medium and 5 mM EDTA in PBS were passed through the column to recover the residual cells. The number of cells in each fraction was determined as described previously.

### 2.13 Analysis of Separated Cells Using Flow Cytometry

Cells separated using the developed PC_7_P_2_M_1_–CD44BP‐modified column were analyzed for the quantitative expression of CD44 and CD105 surface antigens by flow cytometry. The eluate collected from the column was fractionated into 10 mL portions and centrifuged. The resulting cell pellets were washed with phosphate buffered saline (PBS) and fixed in 4% paraformaldehyde (PFA) for 20 min. After removing the PFA solution, nonspecific protein adsorption was blocked using a blocking buffer. Subsequently, the cells were incubated in blocking buffer containing CD105 antibody (1:150; CD105 (Endoglin) Monoclonal Antibody (SN6), PE; eBioscience, Thermo Fisher Scientific Inc.) and CD44 antibody (1:200; Anti‐Human/Mouse CD44 FITC; eBioscience, Thermo Fisher Scientific Inc.) for 2 h. After thoroughly washing with PBS containing 0.05% Tween‐20, the cell suspension was adjusted to a final concentration of 2 × 10^5^ cells/mL (1 mL per sample).

Flow cytometric analysis was performed using a FACS Canto II flow cytometer (BD Biosciences, Franklin Lakes, NJ, USA). Side and forward scatter signals were used to identify cell populations, with unstained HEK293 cells and hMSC serving as negative controls. The fluorescence intensity settings were established using positively stained cell populations (CD44 and CD105). The fluorescence profiles of the separated cell populations were analyzed using FACSDiva 6.1 software (BD Biosciences).

### 2.14 Evaluation of Multipotency of Separated Cells

To evaluate the proliferative and differentiation potential of hMSCs after separation, the cells were subjected to proliferation assays and induced to differentiate into osteoblasts and adipocytes. Their behavior was compared with that of hMSCs before separation.

Cells collected from the PC_7_P_2_M_1_–CD44BP‐modified column and conventionally cultured hMSCs were seeded at a density of 1.0 × 10^4^ cells/cm^2^ and cultured at 37°C in a humidified atmosphere containing 5% CO_2_. The proliferative capacity was assessed by counting the number of viable cells using a hemocytometer on days 1, 2, 3, and 4 after seeding.

For differentiation analysis, both separated and conventionally cultured hMSCs were seeded at a density of 2.0 × 10^4^ cells/cm^2^ and cultured under the same conditions until they reached confluence. The medium was then replaced with the osteogenic or adipogenic differentiation induction medium compositions shown in (Table 3). After 20 days of culture, osteogenic differentiation was qualitatively assessed by alkaline phosphatase staining (Alkaline Phosphatase Live Stain, Invitrogen, Thermo Fisher Scientific Inc.), and adipogenic differentiation was evaluated by Oil Red O staining for lipid droplet formation (FUJIFILM Wako Pure Chemical Co. Ltd.).

**TABLE 3:** Composition of media for osteogenic and adipogenic differentiation.

Osteogenic differentiation	Concentration
FBS	10%
Penicillin–streptomycin 1%	1%
Dexamethasone	100 nM
L‐Ascorbic acid 2‐phosphate trisodium salt	50 μM
*β*‐Glycerophosphate disodium salt n‐hydrate	10 mM
**Adipogenic differentiation**	
FBS	10%
PC/SM	1%
Dexamethasone	1 μM
Indomethacin	0.2 mM
3‐Isobutyl‐1‐methylxanthine	0.5 mM
Insulin	10 μg/mL

### 2.15 Statistical Analysis

Statistical analyses of the contact angle, protein adsorption, and cell adhesion were performed using JMP Pro 15 software (SAS Institute Inc., Tokyo, Japan). One‐way analysis of variance (ANOVA) was conducted to determine the statistical significance among groups. Tukey's honest significant difference test was subsequently applied for multiple comparisons, with the significance level set at *p* < 0.001.

## 3 Results and Discussion

### 3.1 Characterization of Ternary Polymer

In this study, the composition of MPTMS was fixed at 10% for all polymer formulations (PC*_m_* P*_n_* M_1_), and polymers with molecular weights ranging from ≈20,000 to 40,000 were successfully synthesized. Our research group has previously demonstrated two key findings: (1) a surface coating with high coverage can be achieved when the MPTMS content is maintained at 10% and the molecular weight is between 20,000 and 40,000, and (2) increasing the MPTMS content or producing polymers of higher molecular weight leads to gelation during synthesis, as reported in reference [10]. Therefore, these conditions were considered optimal in the present study.

The combination of the three monomers used in this study was intentionally selected to integrate their antifouling properties (CMBMA), peptide conjugation capability (PGMA), and substrate‐anchoring functionality (MPTMS), which together enabled the formation of a stable and selective surface for hMSC capture. We attempted to determine the copolymer composition of various PC*_m_* P*_n_* M_1_ samples using 1H NMR spectroscopy. However, the characteristic proton peaks of each monomer unit overlapped, making an accurate analysis impossible. Consequently, the compositional variations between the CMBMA and PGMA units were evaluated indirectly by preparing polymer‐modified surfaces and analyzing the changes in the elemental composition using XPS (discussed later). The molecular weights obtained from the GPC analysis ranged from ≈20,000 to 40,000 for all polymers. A slight decrease in the molecular weight was observed with increasing PGMA content, which is likely attributable to the hydrophobic nature of the PGMA monomers, limiting their copolymerization efficiency with the hydrophilic monomers CMBMA and MPTMS. A comparison of the feed and obtained monomer ratios revealed that the PGMA content in the final copolymer was slightly lower than that in the initial feed, suggesting incomplete incorporation of PGMA into the polymer structure.

### 3.2 Characterization of the CD44 Binding Peptide

**FIGURE 1:**
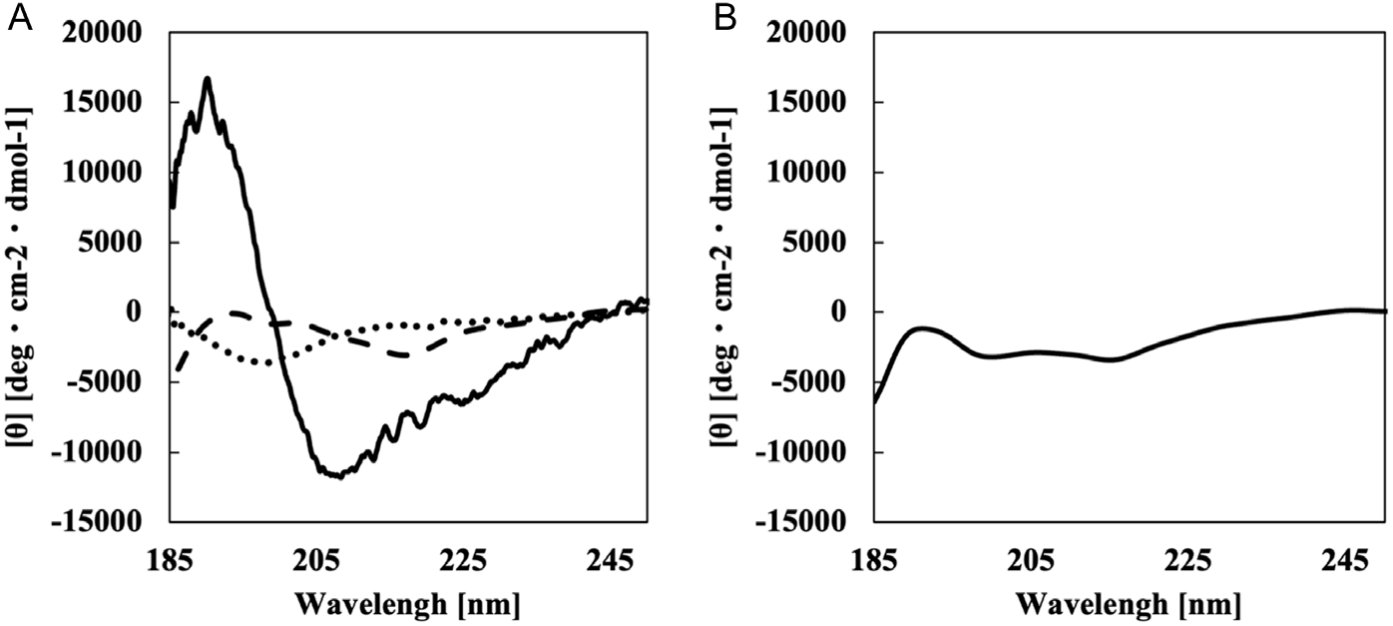
Circular dichroism (CD) spectra of CD44BP peptides. (A) CD spectra of CD44BP(L0)–Aha (solid line), CD44BP(L3)–Aha (dashed line), and CD44BP(L14)–Aha (dotted line). (B) CD spectrum of CD44BP(L3) without Aha modification.

The synthesized CD44BP–Aha and CD44BP peptides were characterized by CD spectroscopy (Figure 1). Upon comparison of CD44BP(L0)–Aha, CD44BP(L3)–Aha, and CD44BP(L14)–Aha (Figure 1A) with CD44BP(L3) (Figure 1B), distinct negative Cotton peaks were observed around 216 nm, indicating the presence of β‐strand and β‐sheet conformations in these peptides. Additionally, CD44BP(L3) exhibited a negative Cotton peak near 200 nm, whereas in CD44BP(L14)‐Aha, the negative Cotton peak near 215 nm disappeared, leaving only a feature around 200 nm. Based on these characteristic spectral features and their relative intensities, it can be inferred that CD44BP(L0)–Aha, which lacks a linker sequence, tends to adopt β‐strand and β‐sheet conformations, whereas the introduction of a linker sequence promotes the transition toward random coil structures.

The molecular weights of the synthesized CD44BP peptides were further evaluated by LC–MS (Figure S2). The theoretical molecular weights of the peptides were as follows: CD44BP(L0)–Aha, 887.57 g/mol; CD44BP(L3)–Aha, 1073.14 g/mol; CD44BP(L14)–Aha, 1784.48 g/mol; and CD44BP, 947.39 g/mol. The measured molecular weights of all the peptides closely matched their theoretical values, confirming their successful synthesis. Additionally, peptide purity was calculated from the peak areas in the LC–MS spectra, which revealed purities greater than 95% for all samples. These findings confirmed that highly pure peptides were successfully obtained.

### 3.3 Surface Characterization of the CD44BP‐Carried Ternary Polymer Surface

For the surfaces modified with PC*_m_* P*_n_* M_1_, the CMBMA content was evaluated by elemental analysis using XPS based on the carbon‐to‐nitrogen (C/N) ratio. Denoting the proportion of CMBMA as “*a*” and the combined proportion of PGMA and MPTMS as “*b*”, Equation (2) was applied to PC_9_P_0_M_1_, while Equation (3) was used for the other copolymers.

(2) $$\frac{C}{N}=\frac{120a+120b}{14a}$$

(3) $$\frac{C}{N}=\frac{120a+204b}{14a}$$

**FIGURE 2:**
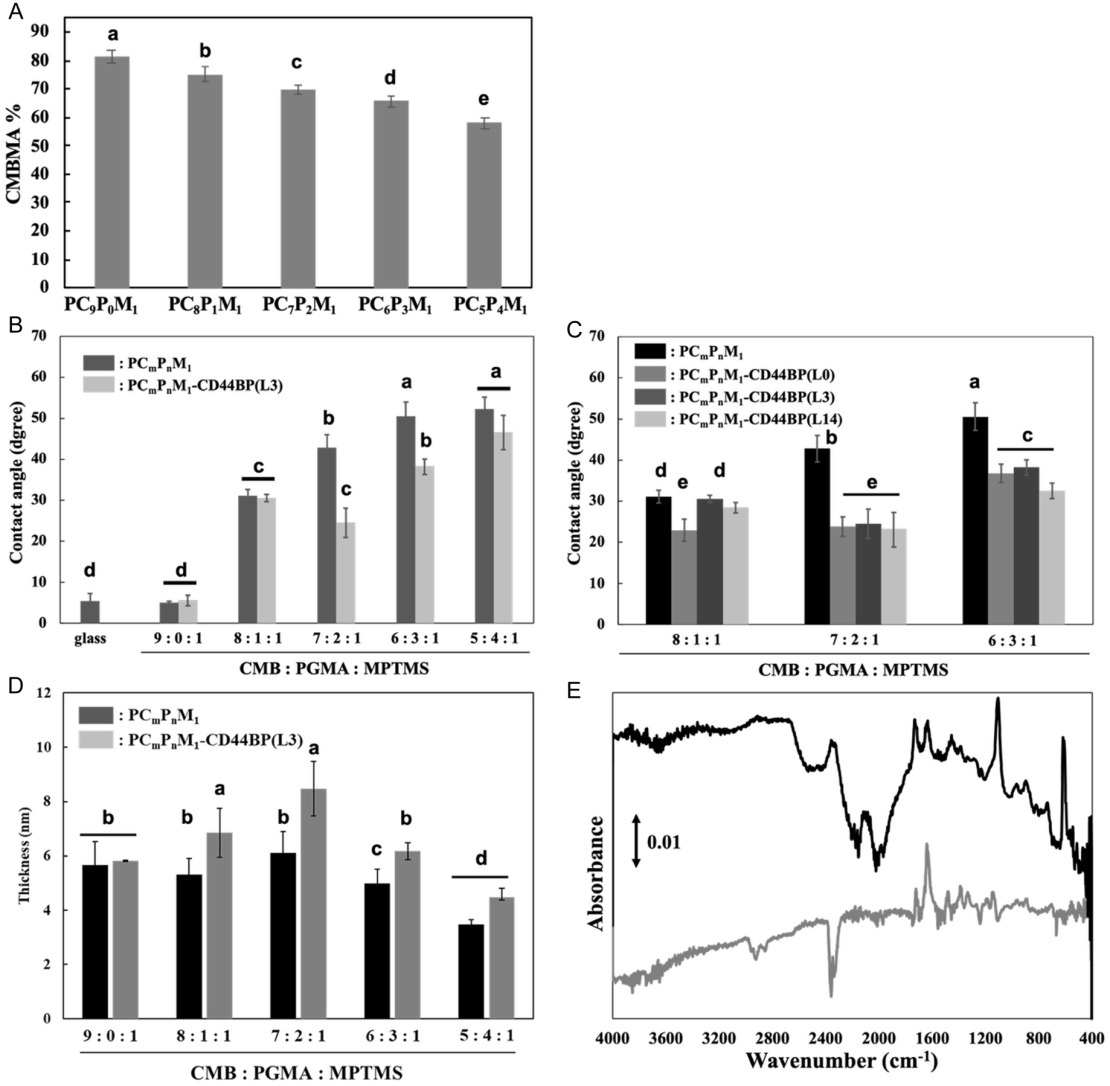
Surface characterization of PC*_m_* P*_n_* M_1_ and PC*_m_* P*_n_* M_1_–CD44BP coatings. (A) Ratio of CMBMA incorporated into PC*_m_* P_*n*_M_1_ copolymers immobilized on the surface. (B) Static water contact angles measured on surfaces modified with PC*_m_* P_*n*_M_1_ (dark gray) and PC*_m_* P_*n*_M_1_–CD44BP(L3) (light gray). (C) Static water contact angles measured on PC_8_P_1_M_1_, PC_7_P_2_M_1_, and PC_6_P_3_M_1_ surfaces functionalized with CD44BP peptides containing different linker lengths (L0, L3, L14). (D) Film thicknesses of surfaces coated with PC*_m_* P_*n*_M_1_ (black bars) and PC*_m_* P_*n*_M_1_–CD44BP(L3) (gray bars). (E) IR spectra of PC_7_P_2_M_1_‐modified surfaces before (black line) and after (gray line) CD44BP(L3) immobilization. Data presentation and statistics: Data in panels (A–D) are presented as mean ± SD (*n* = 3 for A and D; *n* = 10 for B and C). Statistical significance was evaluated using one‐way ANOVA followed by Tukey's HSD test (*p* < 0.001). Bars labeled with different letters indicate statistically significant differences among samples.

The CMBMA contents determined from the C/N ratios of the various PC*_m_* P*_n_* M_1_‐modified surfaces are shown in Figure 2A. The CMBMA content decreased as the initial feed ratio of CMBMA decreased. However, the obtained values deviate slightly from the theoretically expected CMBMA compositions. This deviation can be attributed to the influence of carbon originating from the methoxy groups in MPTMS, as the polymer attachment to the glass substrate was achieved via silane coupling through MPTMS. Consequently, fluctuations in the carbon content of MPTMS likely affected the C/N ratio. Although precise quantification of the CMBMA content and copolymer composition was not possible from these measurements, the results clearly demonstrated the successful fabrication of polymer‐coated surfaces with varying ratios of CMBMA and PGMA. Therefore, these polymer variations enabled further exploration of the optimal surface conditions for selective cell capture. In addition to these observations, it is important to acknowledge the methodological limitations associated with determining copolymer composition. First, 1H NMR analysis could not accurately quantify the amounts of CMBMA, PGMA, and MPTMS because the characteristic peaks of each monomer unit strongly overlapped, preventing precise peak assignment. Second, compositional estimation based on XPS also has intrinsic constraints; the carbon signals derived from the methoxy groups of MPTMS contribute to the overall C/N ratio, causing systematic deviations from the theoretical feed composition and preventing accurate quantification of PGMA, which serves as the peptide anchoring point. Despite these quantitative limitations, this issue did not undermine the interpretation of the overall polymer behavior. Importantly, multiple independent surface analyses, including XPS elemental composition, water contact angle measurements, ellipsometric film thickness, and ATR–FTIR spectra, revealed trends that were consistent with the expected variations resulting from the initial feed ratios of CMBMA and PGMA. These results strongly supported the idea that adjusting the feed ratio effectively modulates the surface properties and peptide‐anchoring capability of the copolymer. Therefore, the selection of PC_7_P_2_M_1_ as the optimal formulation is justified, even though NMR‐ and XPS‐based quantification cannot provide exact compositional values.

Next, the wettability of the PC*_m_* P*_n_* M_1_‐ and PC*_m_* P*_n_* M_1_–CD44BP‐modified surfaces was assessed using static water contact angle measurements (Figure 2B). On the PC*_m_* P*_n_* M_1_‐modified surfaces, the contact angle increased as the CMBMA fraction decreased and the PGMA fraction increased, reflecting a reduction in surface hydration associated with a lower zwitterionic content and a higher proportion of alkyne‐bearing PGMA units. Upon CD44BP(L3) conjugation, the surfaces became more hydrophilic, with the most pronounced decrease in the contact angle observed for PC_7_P_2_M_1_–CD44BP and PC_6_P_3_M_1_–CD44BP. This increase in wettability is consistent with the intrinsic hydrophilicity of the peptide, which includes polar residues such as glutamine. Notably, the extent of the decrease in the contact angle depended on the copolymer composition; PC_8_P_1_M_1_–CD44BP exhibited only a minor change, whereas PC_7_P_2_M_1_–CD44BP exhibited a marked enhancement in wettability. Although a higher PGMA fraction provides more alkyne handles for click conjugation, excessive PGMA content (>30%) may promote hydrophobic association and dense packing of the alkyne side chains, which could reduce the accessibility of reactive groups and limit effective peptide presentation. Collectively, these results identified PC_7_P_2_M_1_ as having the optimal balance between surface hydration and conjugation efficiency for CD44BP attachment. To better capture the dynamic interfacial changes after droplet deposition, the time‐dependent contact angles were measured (Figure S3). All polymer‐ and peptide‐modified surfaces exhibited a monotonic decrease in contact angle over time, characterized by an initial rapid drop, followed by a slower relaxation toward a quasi‐equilibrium value. This behavior supports hydration/relaxation processes within the grafted layer (e.g., water uptake and chain rearrangement), in addition to macroscopic spreading, and qualitatively corroborates the wettability trends obtained from the static measurements. Subsequently, the wettability of the surfaces functionalized with CD44BP(L0), CD44BP(L3), and CD44BP(L14) was evaluated using PC_8_P_1_M_1_, PC_7_P_2_M_1_, and PC_6_P_3_M_1_ (Figure 2C). Although differences in linker length were expected to modulate surface wettability, no significant differences in the contact angles were detected among the three CD44BP variants on any copolymer background. Therefore, the impact of linker length was further considered, primarily in the context of cell capture and adhesion performance.

The film thicknesses of various PC*_m_* P*_n_* M_1_‐ and PC*_m_* P*_n_* M_1_–CD44BP(L3)‐modified surfaces were evaluated using spectroscopic ellipsometry (Figure 2D). The film thickness of the PC_9_P_0_M_1_‐modified surface was measured to be 5.67 ± 0.83 nm. Our research group previously evaluated the film thicknesses of similar copolymer‐modified surfaces [10], demonstrating that coating with PC_9_P_0_M_1_ of ≈10,000 g/mol in average molecular weight produced a film thickness of approximately 3 nm. Because the copolymers used in this study possessed molecular weights ranging from ≈20,000 to 30,000 g/mol, the observed increase in the average film thickness was consistent with expectations based on higher molecular weights. Among the PC*_m_* P*_n_* M_1_‐modified surfaces, those coated with PC_9_P_0_M_1_, PC_8_P_1_M_1_, or PC_7_P_2_M_1_ showed no significant differences in film thickness. However, a noticeable reduction in the film thickness was observed for PC_6_P_3_M_1_ and PC_5_P_4_M_1_ as the PGMA content increased. This decrease can be attributed to the lower average molecular weights of these copolymers and the denser molecular packing induced by the hydrophobic interactions among the alkyne groups at higher PGMA compositions. Furthermore, the film thickness increased upon CD44BP(L3) modification compared with the corresponding copolymer surfaces alone. The PC_7_P_2_M_1_‐modified surface exhibited the most pronounced increase in film thickness following CD44BP(L3) conjugation. This trend correlated well with the enhanced hydrophilicity observed upon peptide attachment, suggesting successful and uniform immobilization of CD44BP(L3) on the copolymer surface.

The ATR–FTIR spectra in the range of 500–4000 cm^−1^ for the PC_7_P_2_M_1_‐ and PC_7_P_2_M_1_–CD44BP(L3)‐modified surfaces are shown in Figure 2E, whereas the spectra for the other PC*_m_* P*_n_* M_1_‐ and PC*_m_* P*_n_* M_1_–CD44BP(L3)‐modified surfaces are presented in Figure S4. Absorption bands were observed at ≈1200 and 1700 cm^−1^ for all copolymer‐modified surfaces, corresponding to the stretching vibrations of the O—C═O bonds in the methacrylate backbone and CMBMA side chains. In contrast, the spectra of the PC*_m_* P*_n_* M_1_–CD44BP(L3)‐modified surfaces exhibited additional absorptions near 1100 cm^−1^, attributable to aromatic C—H stretching vibrations of peptide amino acids, and around 1650 cm^−1^, corresponding to the C═O stretching vibrations (amide I band) of the peptide bonds. These characteristic peaks confirmed the successful immobilization of CD44BP on the copolymer‐coated surface. Furthermore, absorption peaks assigned to alkynes appeared between 2100 and 2300 cm^−1^ on PC_6_P_3_M_1_‐modified surfaces. Although the intensities of these peaks decreased after CD44BP(L3) conjugation, they remained clearly detectable, suggesting that the residual alkyne groups persisted at higher PGMA contents [16, 17]. This observation implies that the alkynyl side chains tend to aggregate within the polymer matrix owing to hydrophobic interactions [18, 19, 20], a finding consistent with the film thickness measurements described earlier.

Scanning electron microscopy (SEM) was performed to examine whether the polymer coating (PC*_m_* P*_n_* M_1_) and subsequent peptide conjugation (PC*_m_* P*_n_* M_1_–CD44BP) induced visually discernible changes in surface morphology (Figure S5). At a low magnification (100×; Figure S5A), the bright region at the top of each image corresponds to the cross‐sectional edge of the glass substrate, confirming that the imaging plane and focus were properly aligned, even in the absence of prominent surface structures. Such wide‐field images were intentionally acquired because, for smooth surfaces, high‐magnification images alone can appear similar and may obscure subtle differences in the overall flatness. All the substrates exhibited a uniformly featureless and smooth appearance with no observable cracks, pores, or micron‐scale aggregates. Moreover, neither local contrast variations nor exposed substrate areas were detected on the PC*_m_* P*_n_* M_1_ or PC*_m_* P*_n_* M_1_–CD44BP surfaces, indicating the absence of increased roughness or uncoated defective regions. Consistent with the low‐magnification observations, high‐magnification images (5000×; Figure S5B) revealed no particulate deposits, phase‐separated domains, or roughened structures after PC*_m_* P*_n_* M_1_ coating or CD44BP conjugation. For all the copolymer compositions examined (PC_8_P_1_M_1_, PC_7_P_2_M_1_, and PC_6_P_3_M_1_), the corresponding PC*_m_* P*_n_* M_1_ and PC*_m_* P*_n_* M_1_–CD44BP surfaces displayed similarly smooth morphologies, forming continuous defect‐free thin films. These SEM observations were in good agreement with the film thickness data obtained by spectroscopic ellipsometry (Figure 2D). All the PC*_m_* P*_n_* M_1_ and PC*_m_* P*_n_* M_1_–CD44BP coatings exhibited film thicknesses below 10 nm, and the maximum increase in thickness upon the conversion of PC*_m_* P*_n_* M_1_ to PC*_m_* P*_n_* M_1_–CD44BP was ≈2 nm. For such sub‐10‐nm ultrathin films, pronounced micro‐ to submicron‐scale topographical changes are not expected, which is consistent with the smooth, defect‐free surface morphology observed by SEM. Therefore, the differences in wettability and cellular responses were primarily attributed to changes in surface chemistry, namely, copolymer composition and peptide functionalization, rather than unintended variations in morphology or topography.

The loading of CD44BP onto various PC*_m_* P*_n_* M_1_‐coated surfaces was evaluated using the microBCA method with silica particles whose surfaces were modified with PC*_m_* P*_n_* M_1_ (Table 4). The results showed a general increase in peptide loading with increasing PGMA content. However, the amount of CD44BP(L3) immobilized on PC_6_P_3_M_1_‐modified surfaces did not increase significantly. This may be attributed to the difficulty in introducing CD44BP(L3) when copolymers with high PGMA content allow alkynyl side chains to pack densely through hydrophobic interactions. This interpretation is consistent with the previous contact angle, film thickness, and ATR–FTIR results, which collectively suggest that alkynyl groups are localized within the copolymer film owing to hydrophobic aggregation.

**TABLE 4:** Amount of CD44BP(L3) bound to PC*_m_* P*_n_* M_1_ on the glass surface.

Copolymer type	Amount of CD44BP(L3) (pmol/cm^2^)^a^	Occupied area of CD44BP (nm^2^/molecule)
PC_8_P_1_M_1_	17 ± 6.2	10
PC_7_P_2_M_1_	50 ± 5.4	3.3
PC_6_P_3_M_1_	53 ± 6.2	3.2

a The amount of CD44BP bound to the copolymer surface was determined using the microBCA assay. Silica microparticles modified with PC*_m_* P_*n*_M_1_ were employed in this analysis.

Based on the measured CD44BP(L3) loading, the peptide density was theoretically sufficient to capture cells expressing CD44 antigens. Table 4 summarizes the peptide amount per unit area of the silica particles and the corresponding specific surface area per peptide molecule. Given that the diameter of hMSCs is ≈10–15 μm, it was estimated that each silica particle carries about 5 × 10^7^ CD44BP molecules, based on its cross‐sectional area. Although the receptor densities vary, membrane receptors are generally present at ≈1 × 10^6^ molecules per cell [21]. Therefore, the CD44BP density on the silica particle surface was considered sufficient for effective interactions with CD44 antigens on the cell membrane.

### 3.4 Suppression of Nonspecific Adsorption and Selective Cell Adhesion on CD44BP‐Functionalized Ternary Polymer Surfaces

**FIGURE 3:**
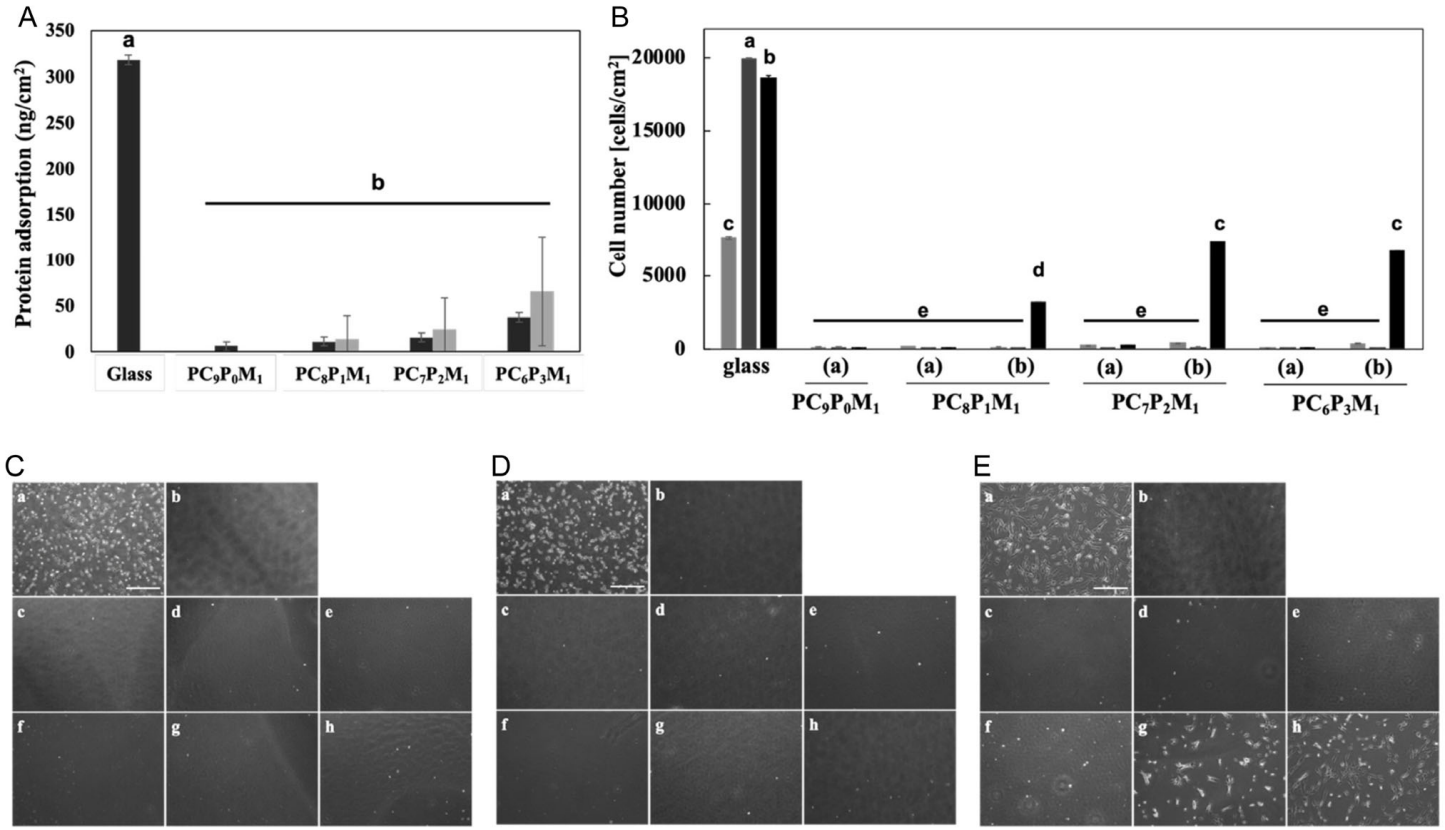
Evaluation of nonspecific protein adsorption and cell adhesion on copolymer‐ and CD44BP(L3)‐modified surfaces. (A) Amount of nonspecifically adsorbed proteins on unmodified glass and on surfaces modified with PC*_m_* P_*n*_M_1_ without peptide (black bars) or PC*_m_* P_*n*_M_1_–CD44BP(L3) (gray bars). Data are presented as mean ± SD (*n* = 10). Statistical significance was evaluated using one‐way ANOVA followed by Tukey's HSD test (*p* < 0.001). (B) Quantitative analysis of cell adhesion on glass, on surfaces modified with PC*_m_* P_*n*_M_1_ without peptide (a), and on surfaces modified with PC*_m_* P_*n*_M_1_–CD44BP(L3) (b) (*n* = 3). NIH3T3 (light gray), HEK293 (gray), and hMSCs (black) were seeded at 2.0 × 10^4^ cells/cm^2^ and cultured for 1 day. Data are shown as mean ± SD. Statistical significance among all samples was assessed using one‐way ANOVA followed by Tukey's HSD test (*p* < 0.001). Bars labeled with different letters represent statistically distinct groups. (C–E) Representative microscopy images of (C) NIH3T3 cells, (D) HEK293 cells, and hMSCs. (E) adhered to surfaces coated with various copolymers: (a) glass, (b) PC_9_P_0_M_1_, (c) PC_8_P_1_M_1_, (d) PC_7_P_2_M_1_, (e) PC_6_P_3_M_1_, (f) PC_8_P_1_M_1_–CD44BP(L3), (g) PC_7_P_2_M_1_–CD44BP(L3), and (h) PC_6_P_3_M_1_–CD44BP(L3). Cells were seeded at 2.0 × 10^4^ cells/cm^2^ and cultured for 1 day. Scale bar: 500 μm.

The nonspecific adsorption of proteins on PC*_m_* P*_n_* M_1_‐ and PC*_m_* P*_n_* M_1_–CD44BP(L3)‐modified surfaces was evaluated (Figure 3A). On all PC*_m_* P*_n_* M_1_‐modified surfaces, the amount of nonspecific protein adsorption was markedly reduced compared to that on the bare glass surface, indicating that the copolymer containing CMBMA effectively suppressed undesired protein adsorption. Moreover, a trend of increased protein adsorption was observed as the CMBMA content decreased. This can be attributed to a reduction in the CMBMA fraction, which is responsible for the antibiofouling properties, combined with the increased hydrophobicity caused by the higher PGMA content.

Conversely, on PC*_m_* P*_n_* M_1_–CD44BP(L3)‐modified surfaces, a relative increase in nonspecific protein adsorption was observed, particularly on the PC_6_P_3_M_1_–CD44BP(L3) surface. This is presumed to result from the lower actual loading of CD44BP(L3) compared to its theoretical value, leaving unreacted PGMA side chains exposed on the surface, which may promote nonspecific protein binding.

Cell adhesion was evaluated on PC*_m_* P*_n_* M_1_‐ and PC*_m_* P*_n_* M_1_–CD44BP(L3)‐modified surfaces. NIH3T3 cells, HEK293 cells, and hMSCs were seeded onto each surface, and after 24 h, both the adhesive morphology and number of adherent cells were analyzed (Figure 3B–E).

On the PC*_m_* P*_n_* M_1_‐modified surfaces, the cell adhesion of NIH3T3, HEK293, and hMSCs was markedly suppressed, as shown in photographs (b)–(e) of Figure 3C–E. These findings are consistent with the protein adsorption results, indicating that the suppression arises from the bioinert nature of the CMBMA units. Conversely, on PC*_m_* P*_n_* M_1_–CD44BP(L3)‐modified surfaces, where bioactive peptides were introduced, the adhesion of NIH3T3 and HEK293 cells remained low, whereas hMSCs exhibited a distinct attachment. Notably, on the PC_8_P_1_M_1_–CD44BP(L3) surface, the number of captured cells was lower than that on the other CD44BP(L3)‐carrying surfaces. This reduction in cell captures was attributed to lower CD44BP loading and higher CMBMA content of the copolymer. Although hMSC adhesion was not significantly different between PC_7_P_2_M_1_ and CD44BP(L3) and PC_6_P_3_M_1_–CD44BP(L3), the reduced antibiofouling effect associated with the unreacted propargyl groups in the PGMA side chains suggests that the PC_7_P_2_M_1_–CD44BP(L3) surface offers optimal conditions for selective hMSC capture. Collectively, these results demonstrated that the fabricated PC*_m_* P*_n_* M_1_–CD44BP(L3) surface selectively captured hMSCs, with PC7P2M1 identified as the optimal copolymer composition for selective cell adhesion.

**FIGURE 4:**
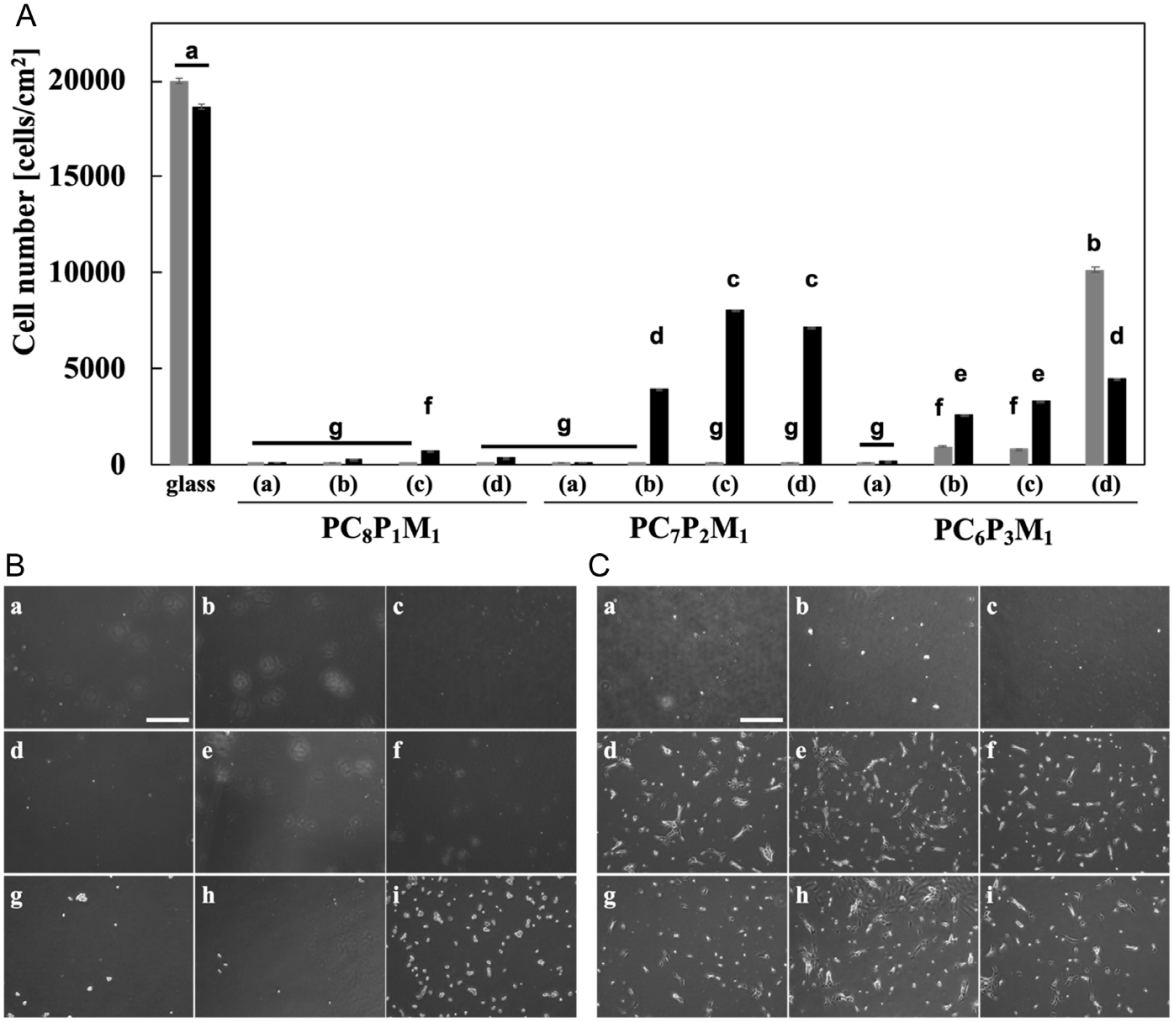
Selective adhesion of hMSCs and HEK293 Cells on copolymer surfaces functionalized with CD44BP of different linker lengths. (A) Quantitative analysis of HEK293 (dark gray bars) and hMSC (black bars) adhesion on surfaces functionalized with CD44BP peptides of different linker lengths (L0, L3, L14) (*n* = 3). Cells were seeded at 2.0 × 10^4^ cells/cm^2^ and cultured for 1 day. For each copolymer composition, the sample labels are as follows: (a) nonpeptide‐modified surface, (b) CD44BP(L0), (c) CD44BP(L3), and (d) CD44BP(L14). Data are presented as mean ± SD (*n* = 3). Statistical significance was evaluated using one‐way ANOVA followed by Tukey's HSD test (*p* < 0.001). Bars labeled with different letters represent statistically distinct groups. (B,C) (B) Representative microscopy images of HEK293 cells and (C) hMSCs adhered to surfaces coated with (a–c) PC_8_P_1_M_1_, (d–f) PC_7_P_2_M_1_, and (g–i) PC_6_P_3_M_1_, functionalized with CD44BP peptides of different linker lengths: (a,d,g) L0, (b,e,h) L3, and (c,f,i) L14. Scale bar: 500 μm.

The adhesion and selective capture of hMSCs were examined using CD44BP peptides with different linker lengths. HEK293 cells and hMSCs were cultured on their respective surfaces, and their adhesion behavior was observed under a phase‐contrast microscope 24 h after seeding (Figure 4).

Quantitative analysis (Figure 4A) showed that HEK293 cell adhesion was effectively suppressed, whereas hMSCs were selectively captured on CD44BP‐modified substrates. Among all the samples, the PC_7_P_2_M_1_‐modified substrate exhibited the highest degree of hMSC adhesion when functionalized with CD44BP(L3). Conversely, the PC_6_P_3_M_1_‐modified substrate showed reduced selectivity, as HEK293 cell adhesion was also observed. This reduced selectivity was attributed to the lower CMBMA content, which diminished the antibiofouling properties of the surface. Meanwhile, the PC_8_P_1_M_1_‐modified surface, containing a higher proportion of CMBMA, exhibited strong suppression of cell adhesion overall but captured fewer hMSCs owing to its lower CD44BP loading.

The results obtained from the PC_7_P_2_M_1_‐ and PC_6_P_3_M_1_‐modified substrates suggest that the amino acid sequence QQGWFP, which specifically interacts with hMSCs, is less exposed on surfaces lacking a linker (L0). Conversely, when the linker was excessively long (L14), the hydrophobic residues within the linker likely caused the hydrophilic glutamine residues at the CD44BP terminus to become embedded within the polymer matrix, thereby reducing the exposure of the bioactive site.

Based on these findings, CD44BP(L3) was identified as the optimal configuration for bioactive peptide presentation, and PC_7_P_2_M_1_ **–**CD44BP(L3**)**‐modified surfaces were selected for use in the subsequent cell‐selective capture device fabrication.

Furthermore, the PC_7_P_2_M_1_–CD44BP(L3)‐modified surface, which demonstrated the highest selectivity for hMSC capture, exhibited approximately 50% reduction in hMSC adhesion compared to the unmodified glass surface. Possible explanations for this reduction include (1) insufficient peptide loading; (2) partial embedding of immobilized CD44BP(L3) within the polymer chains, resulting in a lower effective concentration of accessible peptides compared to total loading; and (3) heterogeneity within the seeded cell population, including cells with reduced CD44 antigen expression.

Regarding point (1), as discussed earlier regarding the antigen density on the cell surface, CD44BP loading on the PC_7_P_2_M_1_–CD44BP‐modified surface (approximately 50 pmol/cm^2^) was estimated to be sufficient for cell capture; therefore, it is unlikely to account for the observed reduction in adhesion. For point (2), no measurement technique is currently available for directly evaluating the structural state of CD44BP within the copolymer matrix; thus, this factor cannot be conclusively confirmed. If a portion of the immobilized CD44BP were buried within the copolymer and rendered nonfunctional, increasing the total peptide loading would have led to an increase in cell adhesion. However, the number of adhered hMSCs on the PC_6_P_3_M_1_–CD44BP‐modified surface, which possessed a higher peptide loading ratio, was nearly identical to that on the PC_7_P_2_M_1_–CD44BP‐modified surface, with only approximately half of the seeded cells adhering. This observation suggests that the maximum feasible amount of CD44BP had already been immobilized on the PC_7_P_2_M_1_–CD44BP‐modified surface and, consequently, that peptide loading was not responsible for the reduction in adhesion. Therefore, point (3) was considered the most plausible explanation for the decreased number of adhered cells. Previous studies have reported that CD44 antigen expression diminishes in hMSCs during differentiation or due to degradation over extended culture periods [22, 23, 24]. Consequently, it is likely that cells exhibiting reduced CD44 expression were not captured by the PC_7_P_2_M_1_–CD44BP‐modified surface, resulting in fewer adhered cells. This hypothesis will be further examined in conjunction with flow cytometry data from capture experiments using cell separation columns.

### 3.5 Selective Cell Separation Using CD44BP‐Functionalized Polymer Columns and Postsorting Evaluation of hMSC Functionality

Based on these findings, the PC_7_P_2_M_1_ **–**CD44BP**‐**modified surface was identified as the optimal configuration for capturing hMSCs expressing CD44 antigens. Therefore, a cell separation column was fabricated by packing silica microparticles modified with PC_7_P_2_M_1_–CD44BP on their surfaces, and the selective separation of hMSCs was investigated. A model cell suspension containing a mixture of hMSCs and HEK293 cells (lacking CD44 antigens) was used to assess separation performance in detail. The eluate was collected in 1 mL fractions, and both cell types and their numbers were quantitatively evaluated.

Silica microparticles with diameters ranging from 250 to 350 μm were selected as the optimal packing material. Preliminary experiments (data not shown) demonstrated that the use of porous silica microparticles allowed protein infiltration, leading to reduced biocompatibility. The 250–350 μm particle size range was found to provide an appropriate balance between contact efficiency and flow rate. Specifically, columns prepared with 75–180 μm particles required ≈2 h for cell separation due to extremely slow elution, while those prepared with 600–850 μm particles exhibited poor separation performance because of excessively rapid elution (within 10 min), which limited cell–surface interaction (data not shown).

In a preliminary examination using a cell separation column packed with nonporous silica beads of 250**–**350 μm in diameter, it was observed that HEK293 cells began to elute ≈6 min after the start of the experiment, with about 80% of the loaded HEK293 cells eluting within 15 min. In contrast, hMSCs began to elute ≈8 min after initiation, and even 25 min after collection, a considerable proportion of hMSCs appeared to remain within the column. Upon subsequent addition of 5 mM EDTA/PBS solution, nearly all the remaining hMSCs were recovered. The fraction eluted with the EDTA solution also contained a small proportion of HEK293 cells. These preliminary findings can be interpreted as follows. The delayed elution of hMSCs is attributed to their specific interaction with the PC_7_P_2_M_1_–CD44BP‐modified silica microparticle surface. The substantial recovery of hMSCs upon treatment with 5 mM EDTA/PBS likely reflects the strong multivalent interactions between CD44 antigens on hMSCs and CD44BP on the polymer‐coated surface. Furthermore, the presence of HEK293 cells in the EDTA‐eluted fraction may be explained by the partial blockage of flow paths caused by the hMSCs bound at multiple contact points, as illustrated in Figure S6, which may have physically trapped the HEK293 cells within the column.

**FIGURE 5:**
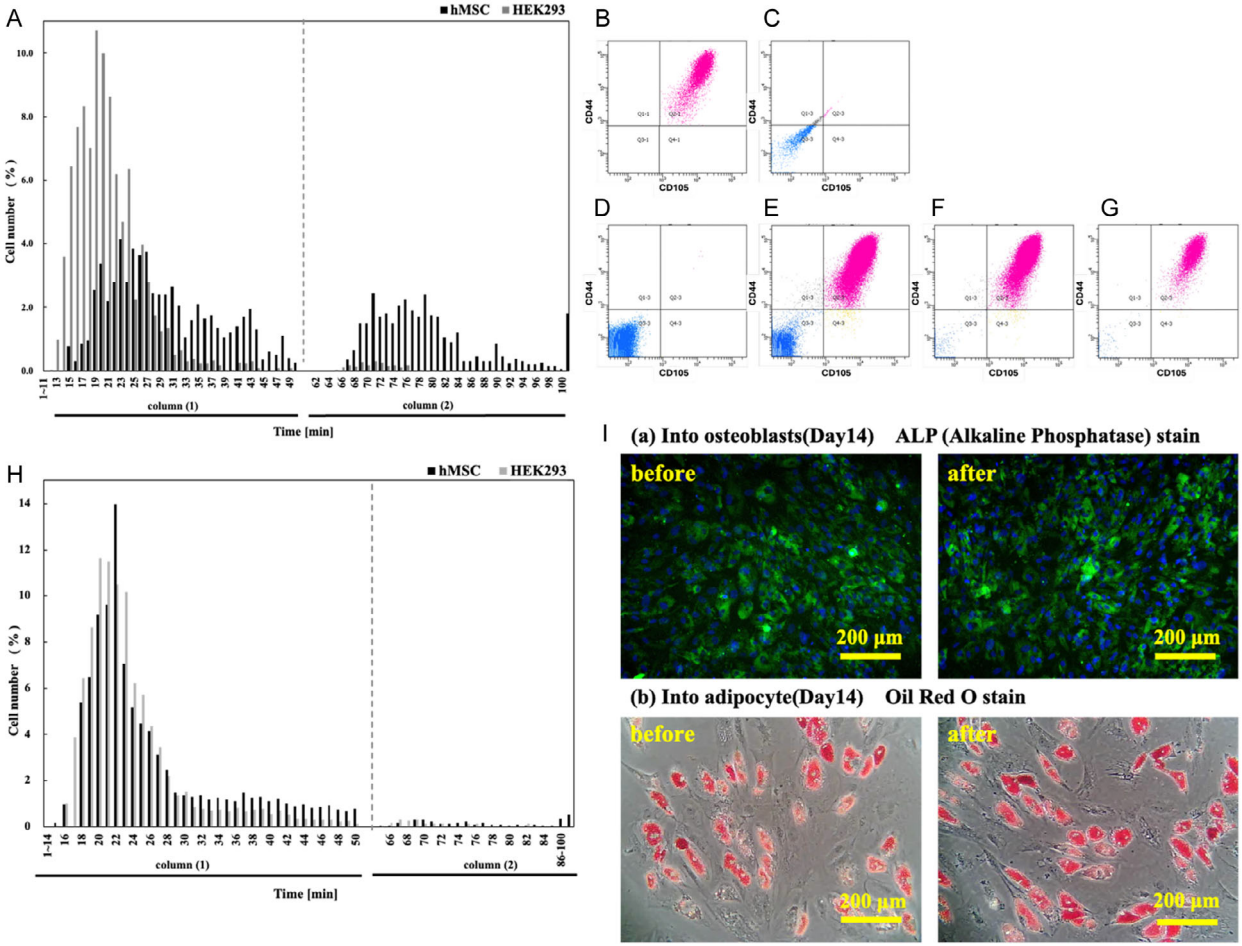
Cell separation performance, CD44BP‐mediated binding specificity, and postsorting differentiation capacity of hMSCs using a PC_7_P_2_M_1_–CD44BP(L3)‐based sorting column. (A) Fractionation profile obtained from a mixed suspension of hMSCs and HEK293 cells using a column packed with PC_7_P_2_M_1_‐CD44BP(L3)‐modified silica beads. The graph shows the number of cells collected in each time fraction based on three independent experiments (*n* = 3). Because the fractions represent continuous, time‐dependent outputs from the column, no statistical analysis was applied to this dataset. (B–G) Flow cytometry analysis of cells before and after column separation. (B,C) Flow cytometry profiles of untreated control (B) hMSCs and (C) HEK293 cells. (D) Fraction collected between 5–15 min. (E) Fraction collected between 16–25 min. (F) Fraction collected between 26–50.5 min. (G) Cells collected after 50.5 min. (H) Fractionation profile obtained when hMSCs were preincubated with excess free CD44BP prior to loading onto the PC_7_P_2_M_1_‐CD44BP(L3)‐modified column. Preincubation with the free peptide competitively inhibited CD44BP‐mediated binding, resulting in a loss of selective retention within the column. Data represent three independent experiments (*n* = 3). Because the fractions are continuous and time‐dependent, no statistical analysis was performed. (I) Evaluation of the multilineage differentiation capacity of hMSCs before and after sorting: (a) Fluorescence micrographs of ALP‐stained osteoblasts. (b) Images of Oil Red O–stained adipocytes.

Therefore, two columns were prepared for the sequential separation experiments. The first column was operated under the same conditions used in the preliminary examination. Twenty minutes after the start of the experiment, all remaining cells retained within the column were recovered by adding 5 mM EDTA/PBS solution. The recovered cells were then washed to remove the residual EDTA and subsequently reintroduced into the column for a second separation (corresponding to the graph *x*‐axis range of 31**–**50.5 min). Finally, 5 mM ethylenediaminetetraacetic acid/phosphate‐buffered saline (EDTA/PBS) was added to elute the remaining bound cells. As shown in Figure 5A, ≈94% of the HEK293 cells were eluted within 15 min from the start of the experiment in the first column, whereas 67% of the loaded hMSCs were contained in the fractions collected between 15 and 25 min and in those eluted with 5 mM EDTA/PBS solution. When the EDTA‐recovered fraction, comprising 51% of the initially added hMSCs and 6% of HEK293 cells, was reapplied to the column, all HEK293 cells eluted within 35 min, and only hMSCs were recovered thereafter.

Although this procedure was not further optimized in the present study, it can be inferred that if high‐purity hMSCs are required, a large quantity of highly pure hMSCs can be obtained by collecting all eluates recovered 15 min after the first separation and performing an additional round of column separation.

The eluate was subsequently divided into groups according to collection time intervals (5–15 min, 16–25 min, 26–50.5 min, and the fraction recovered using 5 mM EDTA/PBS solution), and each group was analyzed by flow cytometry (Figure 5B–G). As a reference, flow cytometric analyses of control hMSCs and HEK293 cells were performed (Figure 5B,C). In the flow cytometry plots, the vertical axis represents the fluorescence intensity corresponding to CD105 antigen expression, whereas the horizontal axis represents the fluorescence intensity corresponding to CD44 antigen expression.

HEK293 cells accounted for 98.7% of the cell population in the fractions collected between 5 and 15 min (Figure 5D). In the fraction collected between 16 and 25 min, 61.6% of the cells were hMSCs coexpressing both CD44 and CD105 antigens, 38.1% were HEK293 cells, and only 0.1% expressed either CD44 or CD105 alone (Figure 5E). In the fraction collected between 26–50.5 min, 98.3% of the cells were hMSCs (Figure 5F), and in the EDTA‐eluted fraction, the hMSC purity reached 99.6%. These results are consistent with those of the quantitative analysis presented in the previous section. Notably, as the elution progressed to later fractions, the double‐positive cell population expressing both CD44 and CD105 exhibited a shift toward higher fluorescence intensities (upward and rightward on the scatter plots). This trend suggests that the hMSCs eluted in the later fractions displayed higher expression levels of CD44 and CD105 antigens. Consequently, these results indicated that the cells collected following repeated column separation possessed enhanced expression of stemness markers, implying a greater degree of maintenance of their undifferentiated state.

In the previous section (specific cell culture on the substrate modified with CD44BP‐anchored ternary polymer), we discussed why only approximately half of the seeded hMSCs adhered to the PC_7_P_2_M_1_–CD44BP‐modified surface. Based on the flow cytometry results, it can be inferred that cells with low CD44 antigen expression failed to adhere to the modified surface. In other words, this separation system not only enables the isolation of specific cell types but also allows the selective capture of hMSCs with high CD44 expression, indicative of a highly undifferentiated state. Since maintaining an undifferentiated state is crucial for efficient lineage‐specific differentiation, a system capable of isolating undifferentiated hMSCs provides a significant functional advantage over conventional separation techniques.

Considering the above findings, it was demonstrated that by introducing a cell suspension into this separation column, collecting the EDTA‐eluted fraction after 15 min, removing the EDTA by centrifugation (≈10 min), and reapplying the cells for a second separation, high‐purity hMSCs could be isolated within 40–50 min. Therefore, this column device represents an efficient, rapid, and low‐damage method for isolating hMSCs, offering a substantial improvement over currently available cell separation approaches.

To confirm that the separation of hMSCs by the PC_7_P_2_M_1_‐CD44BP‐modified column was specifically mediated by the interaction between the CD44 antigen on the cell surface and CD44BP immobilized on the silica particle surface, a competitive inhibition experiment was performed by preincubating the cells with free CD44BP before column loading (Figure 5H). HEK293 cells (gray bars) and hMSCs (black bars) were eluted almost simultaneously, indicating that free CD44BP bound to CD44 antigens on the surface of hMSCs, thereby preventing their binding to CD44BP immobilized on the column. This finding demonstrates that the cell separation mechanism of the PC_7_P_2_M_1_‐CD44BP‐modified column was primarily governed by specific CD44–CD44BP interactions.

The proliferative capacity and maintenance of multipotency of the hMSCs isolated using the constructed separation column (cells collected after 40 min) were evaluated. Proliferative capacity was assessed by determining doubling time, and multipotency was examined by inducing differentiation into osteoblasts and adipocytes.

The doubling time of hMSCs before separation was 40 ± 2.0 h, whereas that of cells after separation was 42 ± 1.5 h. These values are consistent with previously reported doubling times for hMSCs [25, 26, 27, 28, 29], indicating that the separation process using the column does not affect proliferative capacity.

To evaluate the differentiation potential of hMSCs before and after separation, the expression of alkaline phosphatase (ALP), a marker of osteogenic differentiation, was analyzed by fluorescent immunostaining, while lipid droplet formation during adipogenic differentiation was assessed by Oil Red O staining. ALP expression patterns, including the stained area and fluorescence intensity, showed no difference between the pre‐ and postseparation cells (Figure 5I,a). Similarly, the formation of lipid droplets by adipocytes showed no observable differences before and after separation (Figure 5I,b). These findings demonstrated that the osteogenic and adipogenic differentiation abilities of hMSCs were retained following column‐based separation, confirming that the separation procedure did not adversely affect cellular functions.

## 4 Conclusion

A surface functionalized with PC*_m_* P*_n_* M_1_–CD44BP was successfully fabricated, enabling the suppression of nonspecific protein adsorption and nonspecific cell adhesion, while permitting the selective capture of hMSCs, with the PC_7_P_2_M_1_–CD44BP formulation exhibiting the most favorable performance. Based on these optimized surface properties, a cell separation column packed with PC_7_P_2_M_1_‐CD44BP‐modified silica microparticles was constructed, and efficient purification of hMSCs from mixed cell suspensions was demonstrated. Flow cytometry analysis further revealed that hMSCs eluted in the later fractions exhibited high expression of CD44 and CD105, indicating that the separation efficiency was correlated with CD44 expression levels. Notably, the entire separation process was completed within 35 min, and postseparation, hMSCs retained both their proliferative activity and multilineage differentiation capacity. Collectively, these findings demonstrate that this peptide‐functionalized antifouling polymer system enables the rapid, selective, and minimally invasive isolation of high‐quality hMSCs, offering a promising platform for stem cell–based regenerative medicine.

## Supporting Information

Additional supporting information can be found online in the Supporting Information Section. **Supporting Scheme S1:** Schematic illustration of the surface coating process of a glass substrate or silica beads with the CD44BP–functionalized ternary copolymer. **Supporting Scheme S2:** Schematic illustration of silica beads coated with PC_m_P_n_M_1_–CD44BP. **Supporting Fig. S1:** (A) The cell separation column system packed with PC_7_P_2_M_1_–CD44BP–modified silica beads inside a syringe. (B) The procedure for the cell separation experiments. **Supporting Fig. S2:** LC–MS/MS spectra of (A) CD44BP(L3)–Aha and (B) CD44BP(L3). **Supporting Fig. S3:** Time‐dependent water contact angle measurements recorded every 5 s over a 600 s period. Data represent the mean values obtained from five independent measurements. **Supporting Fig. S4:** IR spectra of surfaces modified with PC_m_P_n_M_1_ (black line) and PC_m_P_n_M_1_–CD44BP(L3) (gray line): (A) PC_8_P_1_M_1_, (B) PC_6_P_3_M_1_, and (C) PC_5_P_4_M_1_. **Supporting Fig. S5:** (A,B) SEM images of various polymer‐modified substrates. (A) Images acquired at 100× magnification (scale bar: 500 μm). The bright region at the top corresponds to the cross‐sectional edge of the glass substrate, indicating that the focus is correctly adjusted due to the absence of surface structures. (B) Images acquired at 5000× magnification (scale bar: 10 μm). The examined surfaces were as follows: (a) glass, (b) PC_8_P_1_M_1_, (c) PC_8_P_1_M_1_–CD44BP(L3), (d) PC_7_P_2_M_1_, (e) PC_7_P_2_M_1_–CD44BP(L3), (f) PC_6_P_3_M_1_, (g) PC_6_P_3_M_1_–CD44BP(L3). **Supporting Fig. S6:** Microscopic image of cells within the copolymer–CD44BP–modified column. hMSCs interact with CD44BP on the silica bead surface, leading to partial blockage of the flow path by adhering cells.

## Author Contributions

**Tadashi Nakaji‐Hirabayashi:** conceptualization (lead), data curation (equal), funding acquisition (lead), investigation (equal), methodology (lead), project administration (lead), resources (lead), supervision (lead), visualization (equal), writing – original draft (lead), writing – review and editing (lead). **Moe Kato:** conceptualization (equal), data curation (lead), formal analysis (lead), investigation (lead), methodology (lead), visualization (lead), writing – original draft (lead). **Kazuaki Matsumura:** resources (equal), validation (equal), writing – review and editing (supporting). **Chiaki Yoshikawa:** funding acquisition (equal), methodology (equal), resources (equal), supervision (equal), writing – review and editing (supporting). **Yuki Usui:** funding acquisition (equal), methodology (supporting), resources (supporting). **Takahiro Kishioka:** funding acquisition (equal), methodology (supporting), resources (supporting). **Taito Nishino:** funding acquisition (equal), methodology (supporting), resources (supporting).

## Funding

This Study was supported by the Japan Society for the Promotion of Science (JSPS), Grant‐in‐Aid for Scientific Research (Grant 15H05353, 17KK0130, 18K19907, and 22H3951) and JST SPRING (Grant JPMJSP2145).

## Conflicts of Interest

Usui, Kishioka, and Nishino are employes of Nissan Chemical Corporation. The authors declare no conflicts of interest. Collaboration with Nissan Chemical Corporation did not influence the objectivity of the study or interpretation of the results.

## Supporting information

Supplementary Material

## Data Availability

All data supporting the findings of this study are available in the article and Supplementary Information files.
