# Supplementary material for: CD44‐Binding Peptide‐Functionalized Antibiofouling Polymer Surface for High‐Performance Separation of Human Mesenchymal Stromal Cells
Source: Chembiochem. 2026 Feb 12;27(3):e202500822. doi: 10.1002/cbic.202500822 (PMC12895219; doi:10.1002/cbic.202500822)
Supplement: Supplementary file 1 — Supplementary Material [file CBIC-27-e202500822-s001.pdf]

Supporting Information

CD44-Binding Peptide-Functionalized Anti-Biofouling Polymer Surface for High-Performance Separation of Human Mesenchymal Stromal Cells

Moe Kato[1], *Tadashi Nakaji-Hirabayashi*[1,2,3,5*], *Kazuaki Matsumura*[4], *Chiaki Yoshikawa*[5], *Yuki Usui*[6], *Takahiro Kishioka*[7], *Taito Nishino*[8]

1. *Graduate School of Innovative Life Science, University of Toyama, Toyama 930-0194, Japan*
2. *Faculty of Engineering, Academic Assembly, University of Toyama, Toyama 930-8555, Japan*
3. *Graduate School of Science and Engineering, University of Toyama, Toyama 930-8555, Japan*
4. *School of Materials Science, Japan Advanced Institute of Science and Technology, Nomi, Ishikawa 923-1211, Japan*
5. *Research Center for Functional Materials, National Institute for Materials Science (NIMS), Tsukuba, Ibaraki 305-0047, Japan*
6. *Materials Research Laboratories, Nissan Chemical Corporation.*
7. *Biological Research Laboratories, Nissan Chemical Corporation.*
8. *Head Office, Nissan Chemical Corporation.*

* Corresponding author: T. Nakaji-Hirabayashi, nakaji@eng.u-toyama.ac.jp.

1. Supporting Schemes

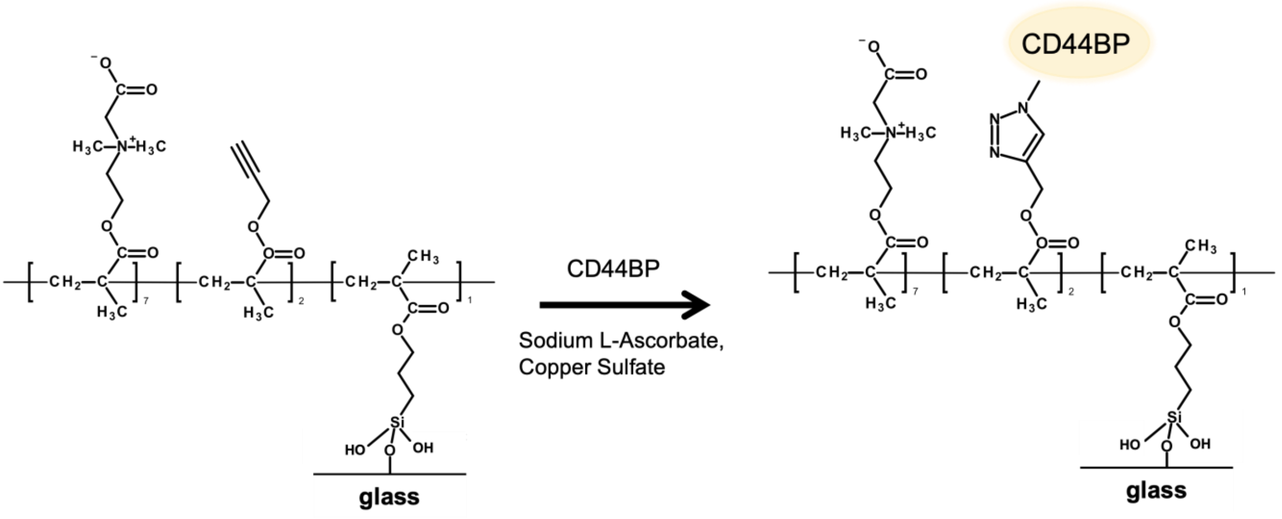

Scheme S1. Schematic illustration of the surface coating process of a glass substrate or silica beads with the CD44BP–functionalized ternary copolymer.

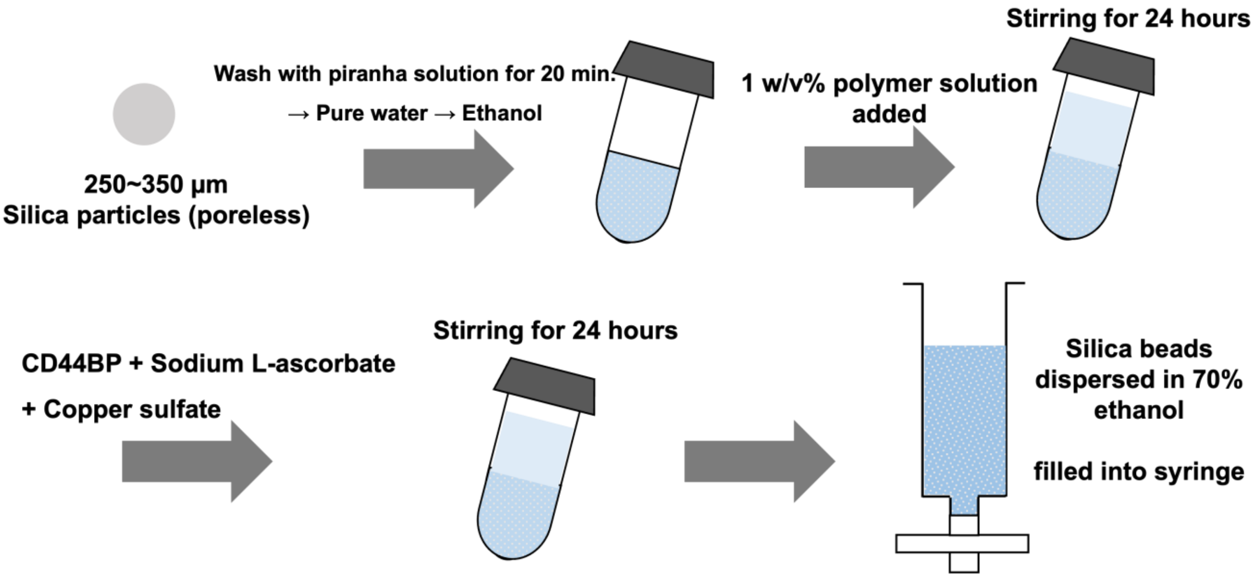

Scheme S2. Schematic illustration of silica beads coated with $PC_mP_nM_1$–CD44BP.

2. Supporting Figures

(A)

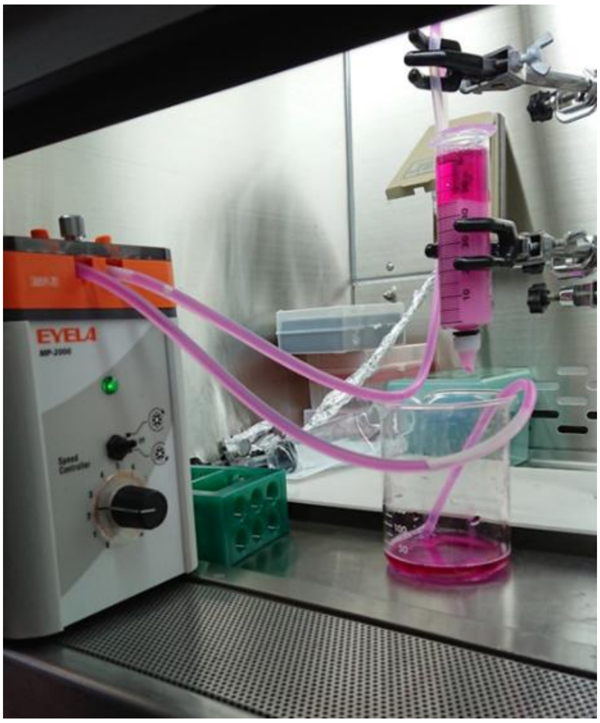

(B)

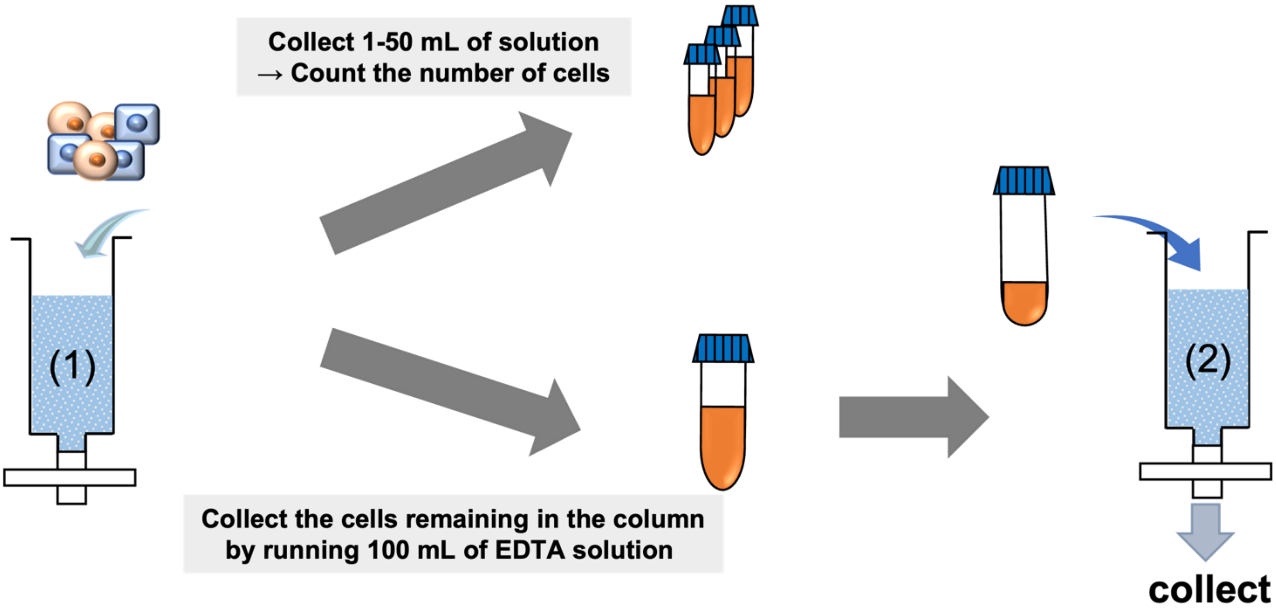

Figure S1. (A) The cell separation column system packed with $PC_7P_2M_1$–CD44BP–modified silica beads inside a syringe. **(B)** The procedure for the cell separation experiments.

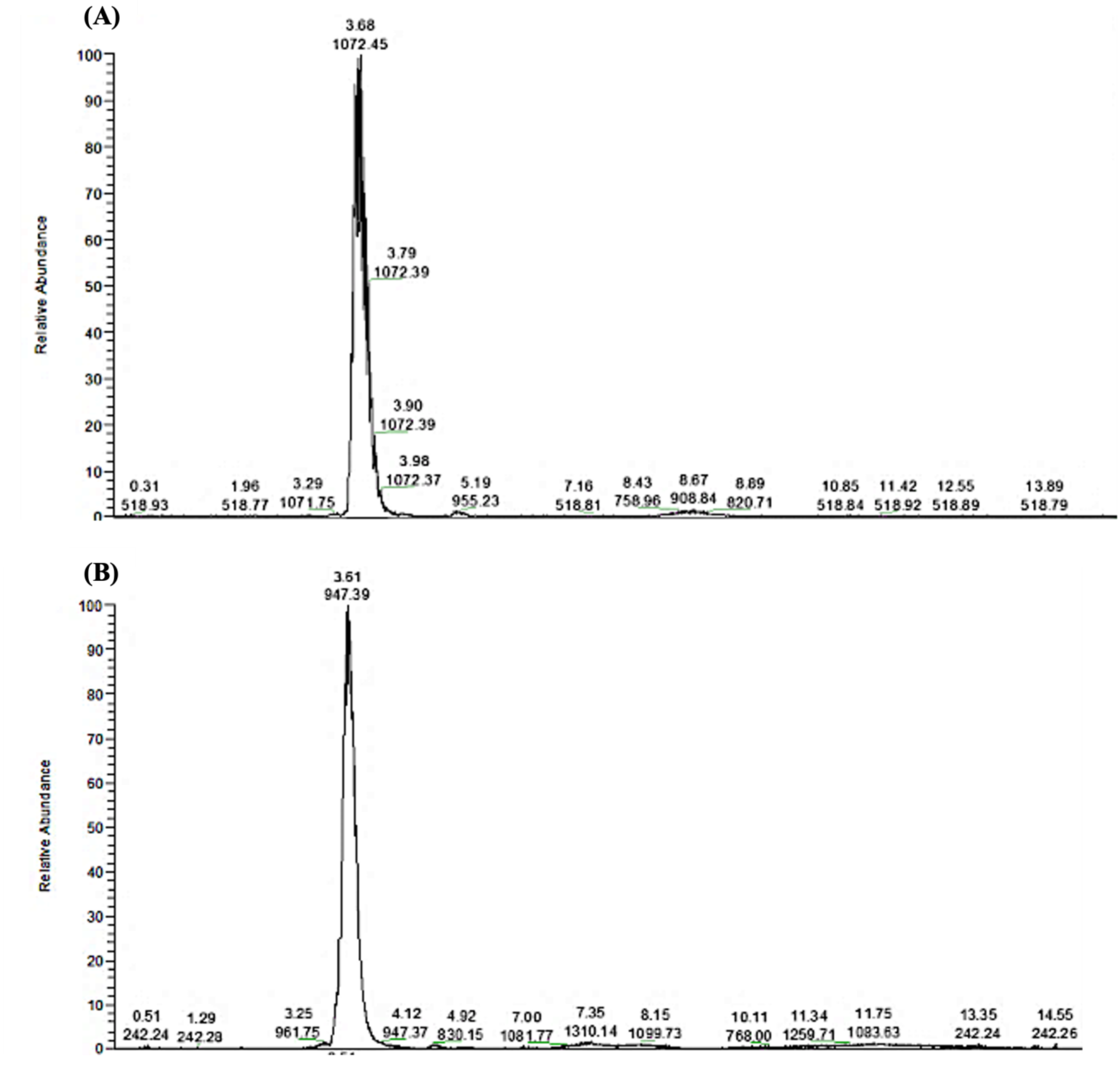

Figure S2. LC–MS/MS spectra of **(A)** CD44BP(L3)**–**Aha and **(B)** CD44BP(L3).

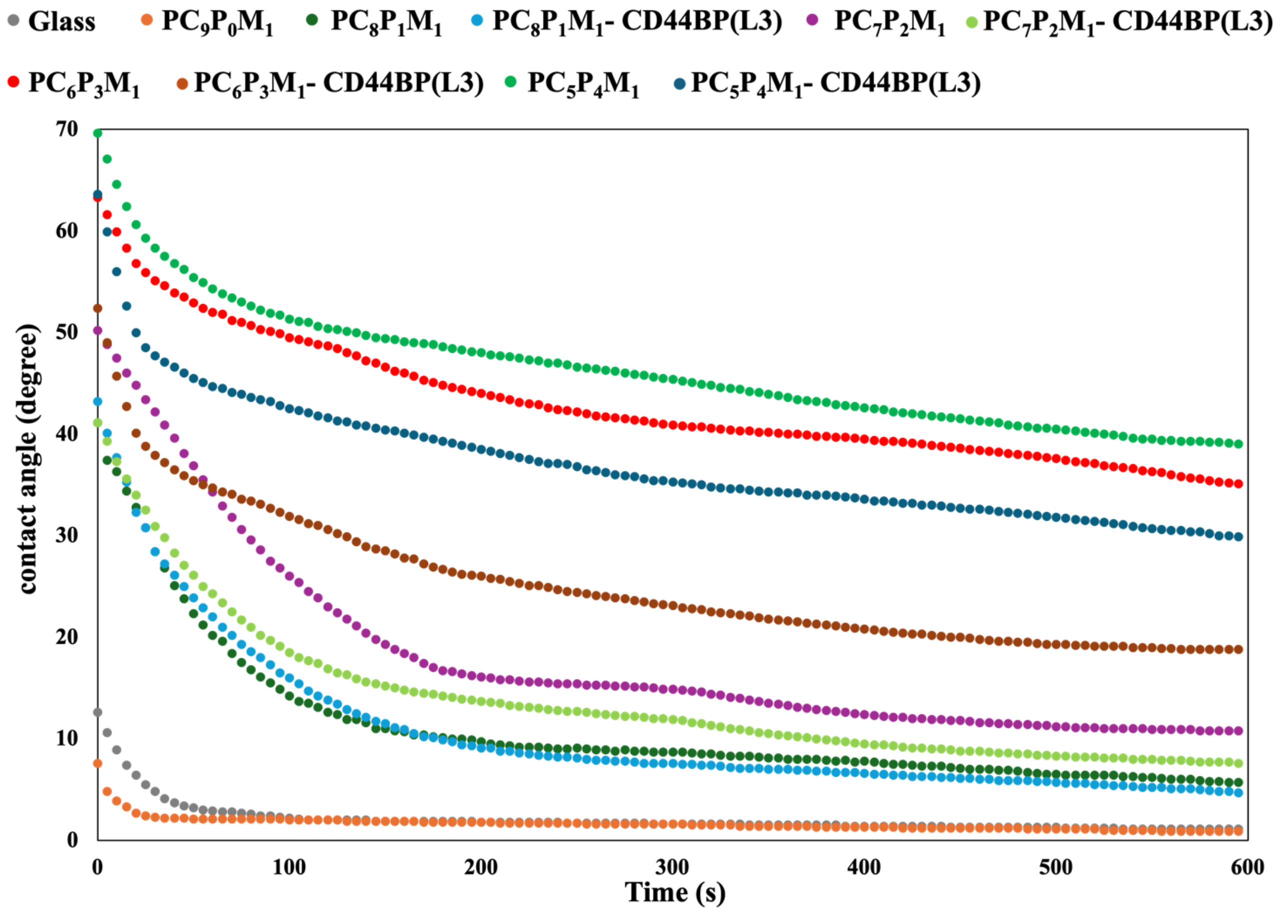

Figure S3. Time-dependent water contact angle measurements recorded every 5 s over a 600 s period. Data represent the mean values obtained from five independent measurements.

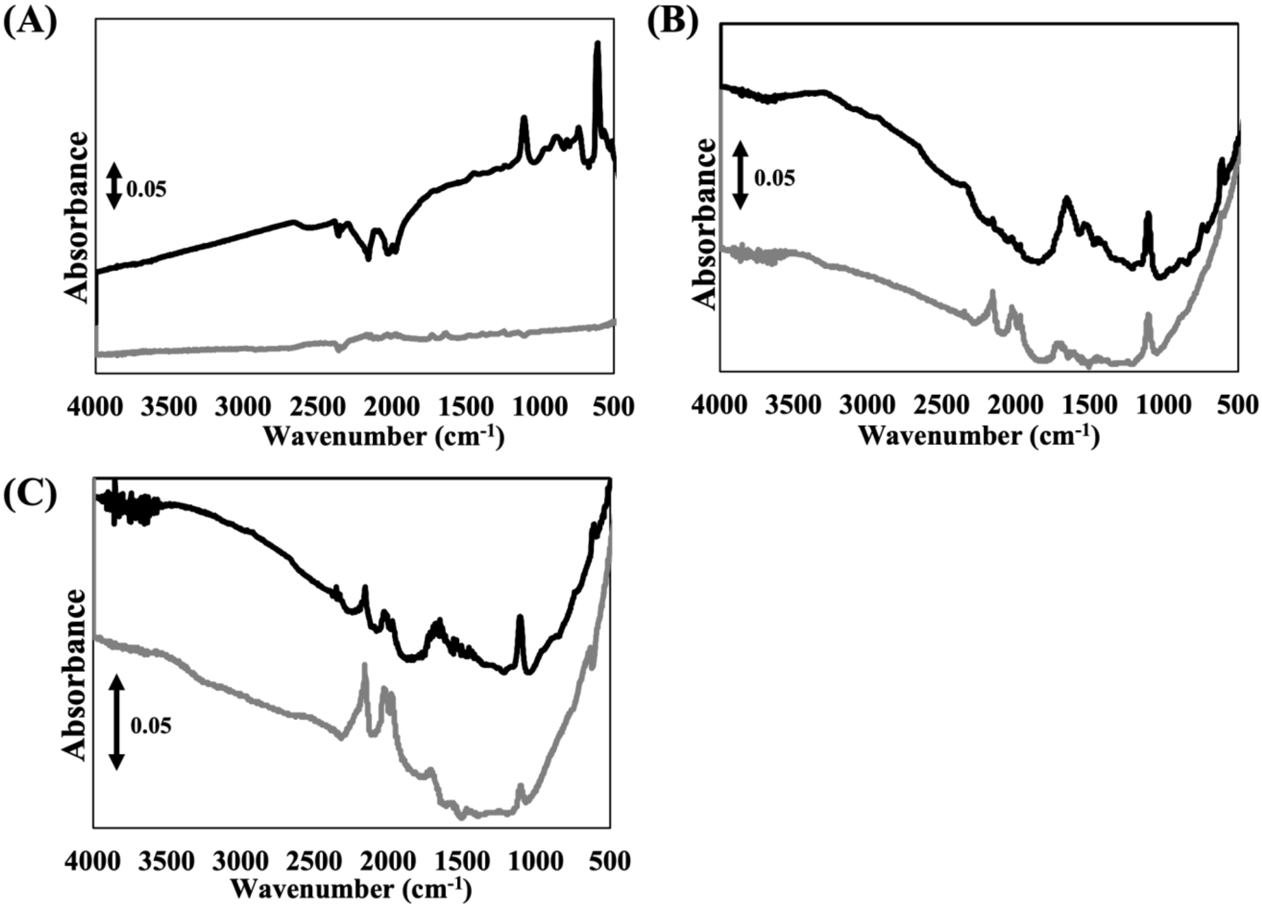

Figure S4. IR spectra of surfaces modified with $PC_mP_nM_1$ (black line) and $PC_mP_nM_1$–CD44BP(L3) (grey line): **(A)** $PC_8P_1M_1$, **(B)** $PC_6P_3M_1$, and **(C)** $PC_5P_4M_1$.

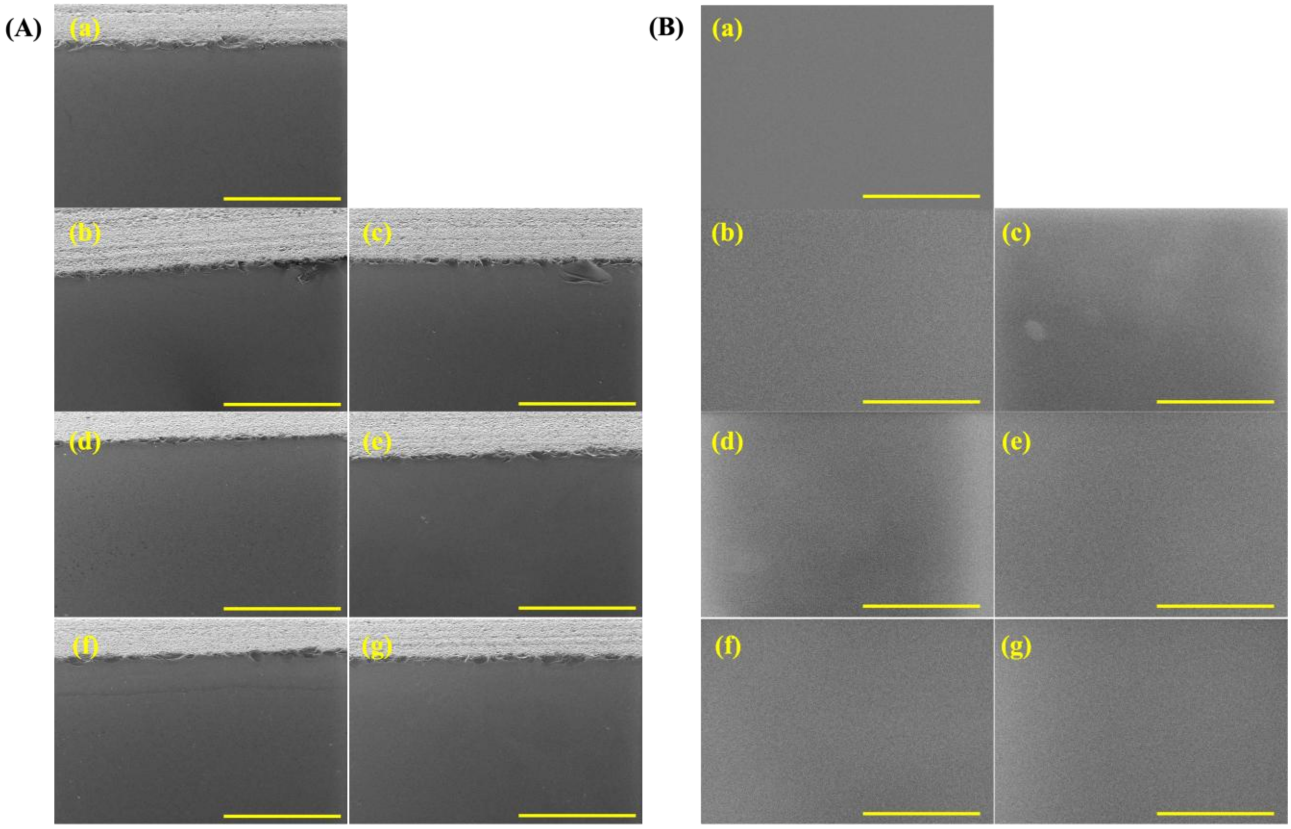

Figure S5. (A, B) SEM images of various polymer-modified substrates. (A) Images acquired at 100× magnification (scale bar: 500 μm). The bright region at the top corresponds to the cross-sectional edge of the glass substrate, indicating that the focus is correctly adjusted due to the absence of surface structures. (B) Images acquired at 5000× magnification (scale bar: 10 μm). The examined surfaces were as follows: (a) glass, (b) $PC_8P_1M_1$, (c) $PC_8P_1M_1$–CD44BP(L3), (d) $PC_7P_2M_1$, (e) $PC_7P_2M_1$–CD44BP(L3), (f) $PC_6P_3M_1$, (g) $PC_6P_3M_1$–CD44BP(L3).

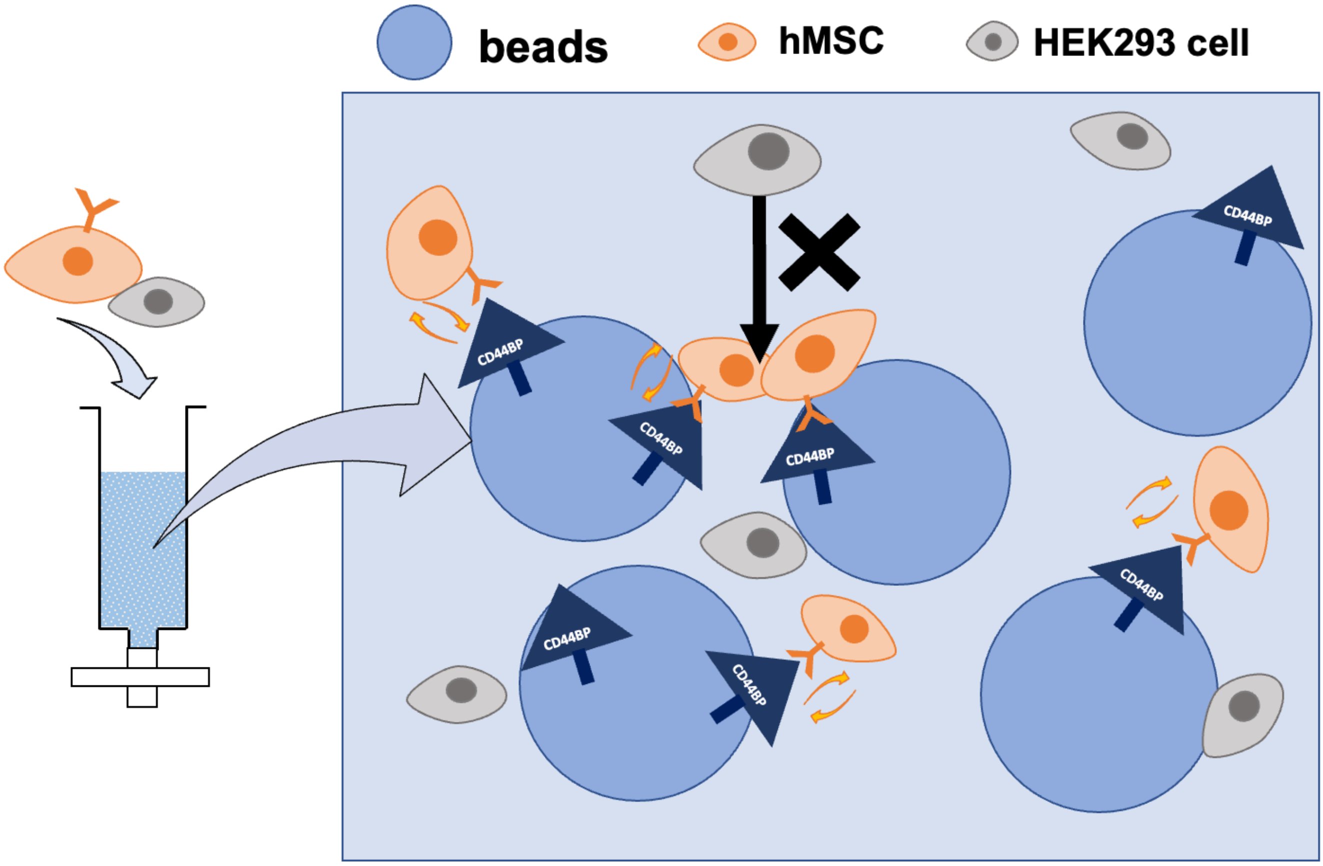

Figure S6. Microscopic image of cells within the copolymer–CD44BP–modified column. hMSCs interact with CD44BP on the silica bead surface, leading to partial blockage of the flow path by adhering cells.
